# Stabilization benefits of single and multi-layer self-nanoemulsifying pellets: A poorly-water soluble model drug with hydrolytic susceptibility

**DOI:** 10.1371/journal.pone.0198469

**Published:** 2018-07-19

**Authors:** Ahmad Abdul-Wahhab Shahba, Fars Kaed Alanazi, Sayed Ibrahim Abdel-Rahman

**Affiliations:** 1 Kayyali Chair for Pharmaceutical Industries, Department of Pharmaceutics, College of Pharmacy, King Saud University, Riyadh, Kingdom of Saudi Arabia; 2 Department of Industrial Pharmacy, Faculty of Pharmacy, Assiut University, Assiut, Egypt; Queen's University at Kingston, CANADA

## Abstract

Solidified self-nanoemulsifying drug delivery systems (SNEDDS) offer strong option to enhance both drug aqueous solubility and stability. The current study was designed to evaluate the potential stabilization benefits of solidifying cinnarizine (CN) liquid SNEDDS into single and multi-layer self-nanoemulsifying pellets (SL-SNEP and ML-SNEP, respectively). The selected formulations were enrolled into accelerated, intermediate and long-term stability studies. The chemical stability was assessed based on the % of intact CN remaining in formulation. The physical stability was assessed by monitoring the *in-vitro* dissolution and physical appearance of the formulations. The degradation pathway of CN within lipid-based formulation was proposed to involve a hydroxylation reaction of CN molecule. The chemical stability study revealed significant CN degradation in liquid SNEDDS, SL-SNEP and ML-SNEP (lacking moisture-sealing) within all the storage conditions. In contrast, the moisture sealed ML-SNEP showed significant enhancement of CN chemical stability within the formulation. In particular, ML-SNEP coated with Kollicoat Smartseal 30D showed superior CN stabilization and no significant decrease in dissolution efficiency, at all the storage conditions. The observed stability enhancement is owing to the complete isolation between CN and SNEDDS layer as well as the effective moisture protection provided by Kollicoat Smartseal 30D. Hence, the degradation problem could be eradicated completely. The incorporation of silicon dioxide had an important role in the inhibition of pellet agglomeration upon storage. Accordingly, ML-SNEP coated with Kollicoat Smartseal 30D and/or silicon dioxide could be an excellent dosage form that combine dual enhancement of CN solubilization and stabilization.

## Introduction

Cinnarizine (CN) is an antihistaminic agent that acts by inhibition of H1 receptor. It is selective calcium ion entry blocker, which selectively acts on arteries. Additionally, it also shows anticholinergic, antiserotonergic, and antidopaminergic activity [1]. CN is a typical BCS class II compound, practically insoluble in water (aqueous solubility < 1μg/ml) with high absorption rate [2, 3]. It is a weak base that shows higher solubility at low pH and lower solubility at high pH [3]. In addition, this drug experiences extensive hydrolytic degradation in aqueous systems [4]. Drug degradation was also observed in pure oils and lipid-based formulations [4, 5].

The optimum method of administering CN has not been fully established, since it suffers poor aqueous solubility as well as chemical instability in aqueous and lipid-based systems [4, 5]. The commercially marketed dosage forms are tablets and capsules. Both of them experience low and erratic oral bioavailability, which is mainly due to the poor and pH-dependent dissolution of the drug [6].

Several studies had attempted to improve the poor-water solubility and low bioavailability of CN [1]. These studies involved using lipid vehicles (such as oleic acid) [7], β-cyclodextrin complex [8], acidifiers [9] and competing agents (such as DL-phenylalanine and L-isoleucine) in combination with β-cyclodextrin complex [10, 11]. Moreover, various dosage forms/delivery systems have been utilized to solve the solubility and bioavailability issues of CN. These include fast dissolving tablets [12, 13], nanoemulsions, microparticles [14], gastroretentive systems [15–17] and self-nanoemulsifying drug-delivery systems (SNEDDS) [6, 18–20]. The fast dissolving tablets showed rapid drug absorption, high drug loading and improved dissolution of the drug. However, they might be limited by having insufficient mechanical strength, hygroscopicity and may lead to unpleasant gritty mouth feeling [1]. The gastro-retentive systems increase the drug residence in stomach which could diminish drug precipitation at alkaline pH and provide sustained effect. However, these systems may cause gastric irritation and the floating tablets/films usually undergo all or none effects [1]. Lipid based systems (such as SNEEDS) enhance the drug gastrointestinal solubilization and resist its precipitation on shifting to higher pH which imply significant bioavailability enhancement. However, these systems suffer from oxidation of unsaturated fatty acids in the formulation and significant drug degradation [1, 4, 5]. Intravenous cinnarizine lipid emulsion was able to show high drug solubility and enhanced chemical stability [4]. However, this system suffers various limitations such as poor physical stability on long-term storage, risk of emboli formation, need for strict aseptic handling and rapid growth of microorganisms [5, 21].

Therefore, it is necessary to design an oral dosage form of CN that could enhance both the aqueous solubility and stability of CN within the formulation. Formulating this drug in SNEDDS could present several solubilization benefits. Recent studies [6, 22] have presented efficient liquid CN-SNEDDS with enhanced aqueous solubility and pH-independent dissolution. The developed SNEDDS maintained high percentage of CN in solution at both pH 1.2 and, most importantly, resisted the significant CN precipitation at pH 6.8. However, such formulations experienced poor physical stability (formulation discoloration) and chemical drug degradation [4, 5]. Solidification of CN liquid SNEDDS, by fluid bed coating, would be a novel approach because it would combine the advantages of liquid SNEDDS and multiparticulate pellets in one dosage form [23]. Our recent studies involved solidification of CN liquid SNEDDS into single-layer self-nanoemulsifying pellets (SL-SNEP) [23] and multi-layer self-nanoemulsifying pellets (ML-SNEP) [24].

**Fig 1:**
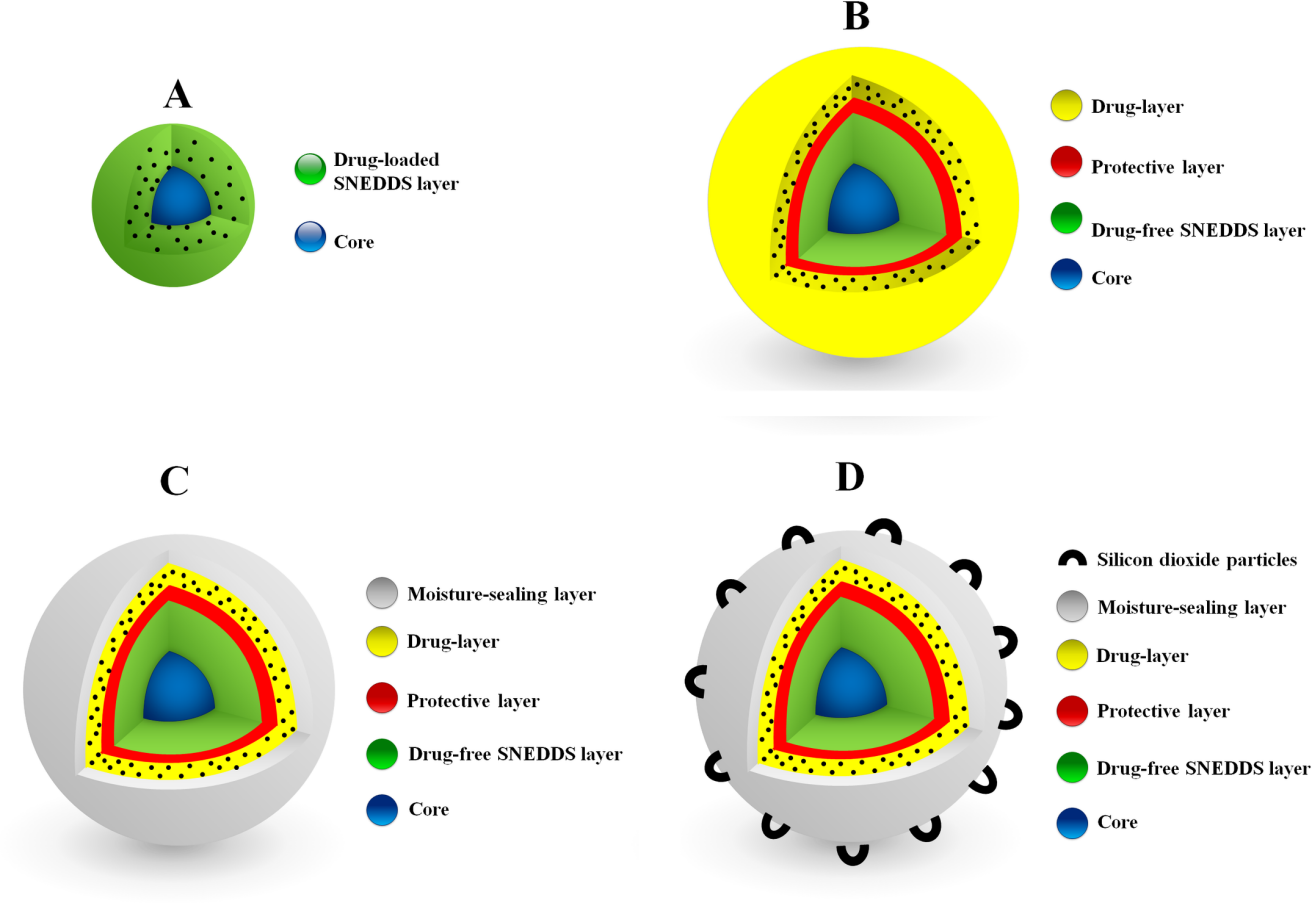
3D schematic diagrams of solid self-nanoemulsifying pellets. (A) SL-SNEP; (B) ML-SNEP1; (C) ML-SNEP2/ML-SNEP3; (D) ML-SNEP2s/ML-SNEP3s. Black spheroids represent CN particles. SNEDDS denote: self-nanoemulsifying drug delivery system.

In SL-SNEP, the drug is in direct contact with the solidified SNEDDS layer (Fig 1A). Therefore, this approach is not expected to stop CN degradation completely because chemical reactions (including drug degradation) would also occur in solid state, but at slower rates [25]. To eradicate the problem completely, CN has to be isolated from solid SNEDDS, during and after manufacturing. To fulfill such requirements, an innovative approach has been recently adopted to prepare the multi-layered self-nanoemulsifying pellets (ML-SNEP) [24]. These ML-SNEP were designed to involve multiple coating layers where the drug-free SNEDDS would be coated in one layer. Another layer would contain the drug without any lipid excipients. To ensure complete isolation, a protective layer was applied between the SNEDDS and drug layers (Fig 1B). Finally, a moisture sealing and anti-adherent layers were optionally applied to protect against drug hydrolysis and pellet adherence, respectively (Fig 1C and 1D). This novel approach is expected to overcome CN degradation in the SNEDDS excipients, enhance the product physical and chemical stability along with maintaining the solubilization benefits of liquid SNEDDS.

In fact, it was necessary to evaluate these two approaches from the stability aspects. The objective of the current study is to assess the chemical and physical stability of CN SL-SNEP and ML-SNEP compared to liquid SNEDDS counterpart. The study involved comprehensive assessment of the formulations stability at accelerated, intermediate and long-term storage conditions. The assessment parameters involved investigating the % of intact CN remaining, *in-vitro* dissolution along with the physical appearance of the formulations. At the end of the study, the two proposed approaches were thoroughly evaluated for their stabilization benefits.

## Materials and methods

### Materials

CN was supplied by FDC Limited (Maharashtra, India). Oleic acid (OL) was purchased from Avonchem, England. Imwitor 308 (I308) was kindly donated by Sasol GmbH (Werk Witten, Germany). Cremophor EL (Cr-El), Kollidon® Polyvinylpyrrolidone (PVP K30) and Kollicoat® Smartseal 30 D were kindly supplied by BASF (Ludwigshafen, Germany). Vcaps Plus® HPMC capsules (size 0) were donated by Capsugel (South Carolina, USA). Non-pareil sugar spheres (850–1000 μm) and Vivapharm® HPMC E3 were purchased from JRS Pharma (Rosenberg, Germany). Plasacryl™T20 was donated by Evonic Industries (Darmstadt, Germany). Butylated hydroxyl toluene (BHT) was purchased from Lobachemie (Mumbai, India). Tributylcitrate (TBC) was donated by Vertellus Performance Materials Inc. (Greensboro, USA).

### CN quantification by UPLC assay

CN was accurately quantified using a validated stability-indicating UPLC reversed-phase method [26], with minor modifications. The mobile phase composition was changed to 0.5% trifluroacetic acid: acentonitrile (55:45) and the run time was increased to 1.5 min, to allow for higher resolution between the intact CN and degradation product peaks. Separation was attained using an Acquity^®^ UPLC BEH C18 (2.1 x 50 mm, 1.7 μm) column along with an Acquity guard filter, maintained at 50°C, and the flow rate was maintained at 0.5 ml/min. The UV detector was set at 251 nm and the injection volume was 1.0 μl.

### Elucidation of degradation pathway by mass spectroscopy

A degraded CN-liquid SNEDDS sample was used to elucidate the drug degradation pathway within lipid-based formulations. The chromatographic separation was performed using UPLC-MS/MS (UPLC: Waters Acquity, Milford, MA, USA). The chromatographic conditions involved the use of C18 column (50 mm×2.1 mm, 1.7μm) along with an Acquity guard filter. The mobile phase comprised 0.5% trifluroacetic acid: acentonitrile (61:39) at a flow rate of 0.25 ml/min. Column temperature was maintained constant at 50°C, the injection volume was 1.0 μl and the run time was 5 min. The eluted compounds were detected by tandem mass spectrometry using TQ detector (Waters Corp., Milford, MA) equipped with an electrospray ionization (ESI) source operating in positive ionization mode. Detection of ionization pairs was performed as follows: CN (m/z 369.2, cone voltage 36V and collision energy 10V), degradation product (m/z 385.2, cone voltage 27V and collision energy 14V) [27].

### Preparation of liquid SNEDDS

Based on the recent optimization studies [28], the liquid SNEDDS was prepared by mixing the components: OL/I308/Cr-El (25/25/50). The formulation components were heated if nesseccary to ensure complete melting and homogeneity. Later, CN was dissolved in the prepared formulations at 80 mg/g concentration. Then, the mixture was thoroughly stirred (at ≈1250 rpm) until the drug is completely dissolved [20]. Finally, the mixtures were filled in air-tight amber glass vials for further investigation [5].

### Preparation of SL-SNEP and ML-SNEP

SL-SNEP and ML-SNEP were prepared using a bottom-spray Mycrolab® fluid bed coater (Oystar Huttlin, Schopfheim, Germany) [23, 24]. Initially, the sugar spheres (850–1000 μm) were charged into preheated fluid bed chamber. For each layer, the coating solution was freshly prepared by mixing appropriate components under mechanical stirring. Then, the coating solution was bottom-sprayed on the fluidizing spheres using a peristaltic pump. After spraying the required solution amount, pellets were dried for at least 10 minutes [23, 29]. The overall composition, formulation and process parameters of SL-SNEP and ML-SNEP are presented in Fig 1 and Tables 1–5.

**Table 1:** Overall composition of various CN self-nanoemulsifying pellet formulations.

Layers	Formulations					
	SL-SNEP	ML-SNEP1	ML-SNEP2	ML-SNEP2s	ML-SNEP3	ML- SNEP3s
**Core**	66.7	61.6	38.4	38.1	38.4	38.1
**Drug-loaded SNEDDS layer**	33.3	-	-	-	-	-
**Drug-free SNEDDS layer**	-	30.8	38.4	38.1	38.4	38.1
**Protective layer**	-	2.8	3.8	3.8	3.8	3.8
**Drug layer**	-	4.8	14.5	14.4	14.5	14.4
**Moisture sealing layer**	-	-	4.8	4.7	4.8	4.7
**Anti-adherent layer**	-	-	-	1.0	-	1.0
**SUM**	100	100	100	100	100	100

SNEDDS: self-nanoemulsifying drug delivery system

**Table 2:** Formulation and process parameters for the preparation of SL-SNEP by fluid bed coating.

Layer	Formulation parameters				Process parameters					
	Coating solution composition	Ratio (w/w%)	Target CWG (%)	Coating solution conc.	Inlet temp (°C)	Product temp (°C)	Inlet air volume (m^3^/h)	SAP (bar)	MAP (bar)	Spray rate (rpm)
Drug-loaded SNEDDS layer	CN	0.6	50	19.6%	60–63	50.53	35	1.2	0.6	4–24
	OL	1.8								
	I308	1.8								
	Cr-EL	3.6								
	HPMC E3	10.7								
	Plasacryl T20	1.1								
	water	80.4								
	***Sum***	***100*.*0***								

SNEDDS: self-nanoemulsifying drug delivery system; CN: cinnarizine; OL: oleic acid; I308: Imwitor308; Cr-El: cremophor El; CWG: coating weight gain; SAP: spray air pressure; MAP: microclimate air pressure.

**Table 3:** Formulation and process parameters for the preparation of ML-SNEP1 by fluid bed coating.

Layer	Formulation parameters				Process parameters					
	Coating solution composition	Ratio (w/w%)	Target CWG (%)	Coating solution conc.	Inlet temp (°C)	Product temp (°C)	Inlet air volume (m^3^/h)	SAP (bar)	MAP (bar)	Spray Rate (rpm)
Drug-free SNEDDS layer	OL	2.0	50	19.6%	55–60	50–53	35–40	1.0–1.5	0.5–0.8	4–6
	I308	2.0								
	Cr-EL	3.9								
	HPMC E3	10.7								
	Plasacryl T20	1.1								
	water	80.4								
	***Sum***	***100*.*0***								
Protective layer	HPMC E3	5.0	3	5%	64–67	38–42	25	1.2	0.6	20–24
	Water	95.0								
	***Sum***	***100*.*0***								
Drug layer	CN	0.7	5	3.5%	41–43	32–34	25	1.2	0.6	6–8
	PVP K30	2.8								
	0.1M HCl	96.5								
	***Sum***	***100*.*0***								

SNEDDS: self-nanoemulsifying drug delivery system; CN: cinnarizine; OL: oleic acid; I308: Imwitor308; Cr-El: cremophor El; PVP: Polyvinylpyrrolidone; CWG: coating weight gain; SAP: spray air pressure; MAP: microclimate air pressure.

**Table 4:** Formulation and process parameters for the preparation of ML-SNEP2 by fluid bed coating.

Layer	Formulation parameters				Process parameters					
	Coating solution composition	Ratio (w/w%)	Target CWG (%)	Coating solution conc.	Inlet temp (°C)	Product temp (°C)	Inlet air volume (m^3^/h)	SAP (bar)	MAP (bar)	Spray Rate (rpm)
Drug-free SNEDDS layer	OL	1.5	100	15%	60–73	50–52	35	1.2–1.4	0.6–0.7	6–28
	I308	1.5								
	Cr-EL	3.0								
	HPMC E3	8.2								
	Plasacryl T20	0.8								
	water	85.0								
	***Sum***	***100*.*0***								
Protective layer	HPMC E3	5.0	5	5%	52–55	38–41	25	1.2–1.4	0.6–0.7	10–14
	Water	95.0								
	***Sum***	***100*.*0***								
Drug layer	CN	0.6	18	3%	43–53	32–35	25–30	1.2–1.4	0.6–0.7	8–18
	PVP K30	2.4								
	0.12M HCL	97.0								
	***Sum***	***100*.*00***								
Moisture sealing layer	HPMC E3	5.0	5	5%	49–56	37–38	25	0.8–1.2	0.4–0.6	6–18
	Water	95.0								
	***Sum***	***100*.*0***								

SNEDDS: self-nanoemulsifying drug delivery system; CN: cinnarizine; OL: oleic acid; I308: Imwitor308; Cr-El: cremophor El; PVP: Polyvinylpyrrolidone; CWG: coating weight gain; SAP: spray air pressure; MAP: microclimate air pressure.

**Table 5:** Formulation and process parameters for the preparation of ML-SNEP3 by fluid bed coating.

Layer	Formulation parameters				Process parameters					
	Coating solution composition	Ratio (w/w%)	Target CWG (%)	Coating solution conc.	Inlet temp (°C)	Product temp (°C)	Inlet air volume (m^3^/h)	SAP (bar)	MAP (bar)	Spray Rate (rpm)
Drug-free SNEDDS layer	OL	1.5	100	15%	60–70	50–52	35	1.2–1.4	0.6–0.7	8–22
	I308	1.5								
	Cr-EL	3.0								
	HPMC E3	8.2								
	Plasacryl T20	0.8								
	water	85.0								
	***Sum***	***100*.*0***								
Protective layer	HPMC E3	5.0	5	5%	51–54	37–40	25	1.2–1.4	0.6–0.7	10–16
	Water	95.0								
	***Sum***	***100*.*0***								
Drug layer	CN	0.6	18	3%	43–52	33–35	25–30	1.2–1.4	0.6–0.7	8–18
	PVP K30	2.4								
	0.12M HCL	97.0								
	***Sum***	***100*.*00***								
Moisture sealing layer	Kollicoat Smartseal 30D	13.0	5	15%	33–42	28–29	25	0.8–1.2	0.4–0.6	4–15
	TBC	1.7								
	BHT	0.3								
	Water	85.0								
	***Sum***	***100*.*0***								

SNEDDS: self-nanoemulsifying drug delivery system; CN: cinnarizine; OL: oleic acid; I308: Imwitor308; Cr-El: cremophor El; PVP: Polyvinylpyrrolidone; TBC: Tributylcitrate (plasticizer); BHT: Butylated Hydroxytoluene (anti-oxidant); CWG: coating weight gain; SAP: spray air pressure; MAP: microclimate air pressure.

#### Preparation of SL-SNEP

SL-SNEP were designed to involve a single coating layer of CN-loaded SNEDDS on top of sugar spheres. The layer constituted 33.3% of the total pellet weight and involved drug-loaded SNEDDS/HPMC E3/Plasacryl T20 at the ratios (40/54.5/5.5) (Fig 1A, Tables 1 and 2) [23].

#### Preparation of ML-SNEP

These pellets were designed to involve multiple (3–5) coating layers where the drug-free SNEDDS layer was completely isolated from CN layer. Accordingly, several multi-layered pellets were prepared by varying the composition and number of coating layers as follows (Fig 1B–1D, Tables 1 and 3–5).

#### Drug-free SNEDDS layer

This layer involved similar excipients to drug-loaded SNEDDS layer, but the SNEDDS was prepared without the incorporation of CN [24]. The layer involved drug-free SNEDDS/HPMC E3/Plasacryl T20 at the ratios (40/54.5/5.5) and constituted 30.8–38.4% of the total pellet weight (Tables 1 and 3–5)

#### Protective layer

This layer was applied on top of the drug-free SNEDDS layer to isolate it from the drug-layer. This layer involved pure HPMC E3 and constituted 2.8–3.8% of the total pellet weight (Tables 1 and 3–5).

#### Drug layer

This layer was applied on top of the protective layer. The drug layer involved CN/PVP k30 at the ratios (20/80) and constituted of 4.8–14.5% of the total pellet weight (Tables 1 and 3–5).

#### Moisture sealing layer

This layer was applied on top of the drug layer (in certain ML-SNEP batches) to protect the drug from exposure to atmospheric moisture (Tables 1, 4 and 5). It constituted 4.7–4.8% of the total pellet weight and involved using two sets of polymers either pure HPMC E3 or Kollicoat smartseal 30D/TBC/BHT at the ratios (87/11/2).

#### Anti-adherent layer

This layer was applied on top of the moisture-sealing layer (in certain ML-SNEP batches) to minimize pellet agglomeration upon storage (Table 1). It involved adding silicon dioxide as a top powder (at 1% w/w) through dry mixing with the pellets.

### Pellet size and size distribution

The average pellet diameter was measured using an EVO LS10 scanning electron microscope (Carl Zeiss; Cambridge, United Kingdom). Samples were gold-coated by Q150R sputter coater (Quorum Technologies Ltd, East Sussex, UK). The process was carried out in an argon atmosphere, at 20 mA for 1 min and involved applying 3–10 KV excitation electron energy [30]. Sieve analysis study was performed for pellet size distribution. To perform the analysis, the pellets were passed through stacks of sieves of 600, 800, 1000, 1250, 1600, 1700 and 2000 μm [31]. A minimum of three replicates was considered for each sample.

### Stability studies

The chemical and physical stability of CN formulations were assessed at accelerated, intermediate and long-term storage conditions. The chemical CN stability was evaluated based on % of intact CN remaining in formulation. To determine the intact CN concentration, liquid SNEDDS samples were directly diluted in acetonitrile and assayed by UPLC [5]. While, solid SNEP were initially crushed using mill, then an aliquot was dissolved in acetonitrile, sonicated for at least 10 minutes and centrifuged [32]. Supernatant (1 ml) was diluted in acetonitrile and assayed by UPLC [26]. A minimum of six replicates were considered for each sample. Furthermore, the *in-vitro* dissolution of each formulation was investigated and compared to initial profile. Finally, the physical appearance of the formulation was examined to record any turbidity, color change or pellet agglomeration.

#### Accelerated stability studies

Both liquid SNEDDS and solid SNEP (filled in air-tight amber glass vials) were stored in climatic stability chambers (KBF-ICH 240/720 series, Binder Gmbh, Tuttlinger, Germany) at 40 **°**C ± 2 **°**C and relative humidity (RH) of 75% ± 5% [33, 34]. Samples were withdrawn after a minimum of 3 and 6 months, then allowed to room temperature prior to investigation.

#### Intermediate stability study

Both liquid SNEDDS and solid SNEP (filled in air-tight amber glass vials) were stored at 30 **°**C ± 2 **°**C and 65% RH ± 5% [33, 34]. Samples were withdrawn at after a minimum of 3 and 6 months.

#### Long-term stability study

Both liquid SNEDDS and solid SNEP (filled in air-tight amber glass vials) were stored at 25 **°**C ± 2 **°**C and 60% RH ± 5% [33, 34]. Samples were withdrawn after a minimum of 3, 6, and 12 months [33].

#### *In-vitro* dissolution studies

*In-vitro* dissolution studies were utilized as a tool to assess the physical stability of liquid SNEDDS, SL-SNEP and ML-SNEP at different storage conditions. The *in-vitro* dissolution studies were conducted using USP dissolution apparatus II (Model: UDT-804, LOGAN Inst. Corp., USA) at 50 rpm paddle stirring rate and 37 ± 0.5 **°**C temperature. All the tested formulations were utilized in amounts equivalent to 25 mg CN. Initially, the dissolution medium was composed of 500 ml 0.1N HCl (pH 1.2). Consecutive samples were withdrawn at 5, 10, 15, 30, 60 and 120 min then centrifuged and assayed by UPLC [6]. After the “120 min” sample, the dissolution medium was adjusted to pH 6.8 by adding 250 ml of 120 mM tribasic sodium phosphate. In the new pH, samples were collected at 15, 30, 60 and 120 min, centrifuged and assayed as mentioned earlier [6]. In case liquid SNEDDS, the formulations were filled into HPMC capsules (size 0) prior to conducting the dissolution studies. A few turns of nonreactive wire-helix were attached to each capsule to prevent its floating [35]. A minimum of three replicates were considered for each sample. The dissolution profiles were characterized using dissolution efficiency (DE) which was calculated from the area under the dissolution curve at time t (determined using the non-linear trapezoidal rule) and expressed as a percentage of the area of the trapezoid described by 100% dissolution in the same time) [36].

### Statistical analysis

QI Macros 2016 and SPSS statistics 25 were utilized to explore the significance of the data. One-way ANOVA followed by post Hoc Tests (LSD) were applied to compare the pellet size, the % of intact CN remaining and DE. Two-way ANOVA was applied to compare the overall chemical stability of the formulations [6, 37]. A value of p < 0.05 was considered significant throughout the study.

## Results and discussion

### Elucidation of degradation pathway by mass spectroscopy

**Fig 2:**
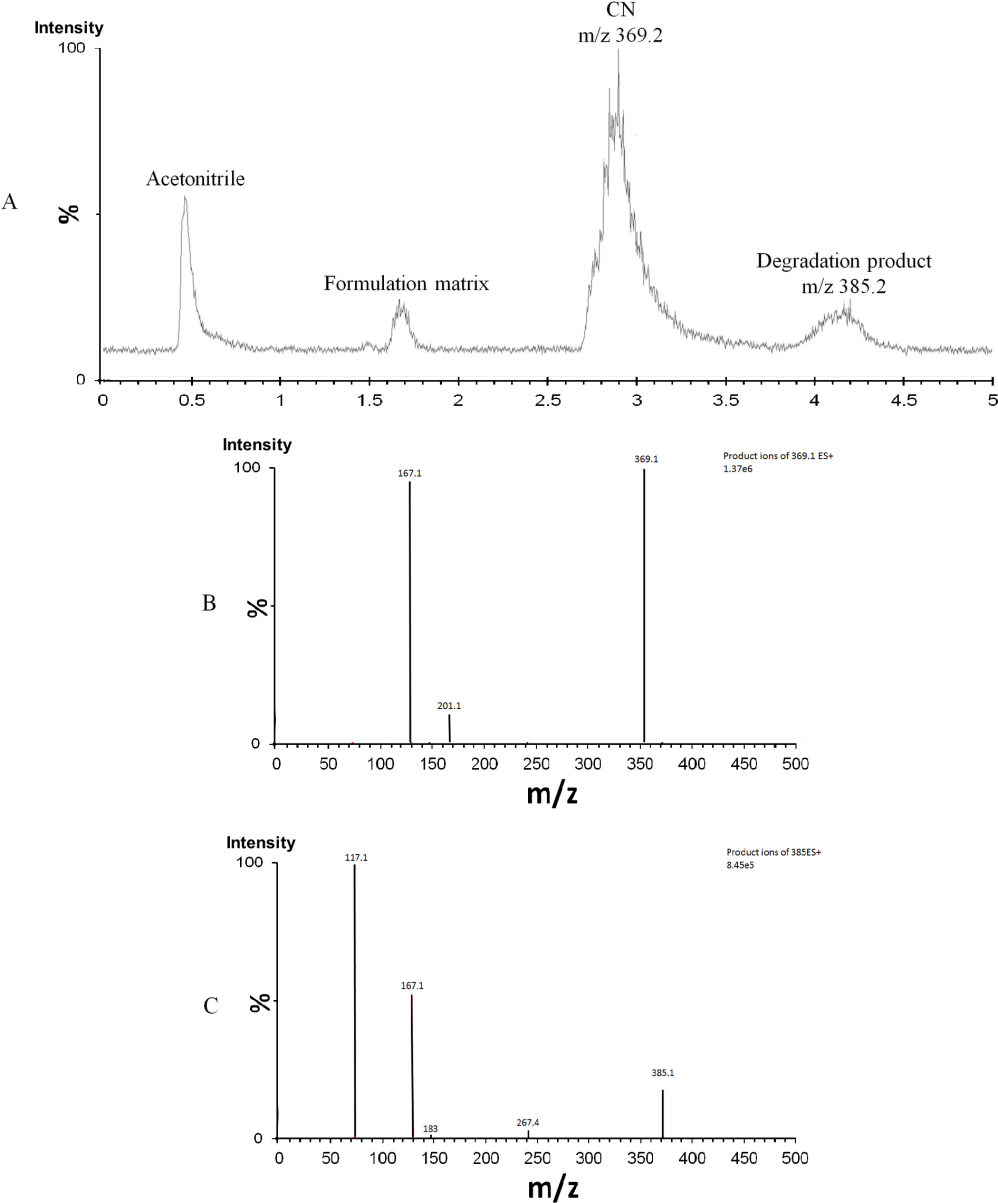
Mass spectrometry of CN and its degradation product within lipid-based formulations. (A) UPLC/MS chromatogram of degraded CN-liquid SNEDDS sample; (B) MS^2^ mass spectrum of [CN]+ ion (m/z 369.2); (C) MS^2^ mass spectrum of [degradation product]+ ion (m/z 385.2).

**Fig 3:**
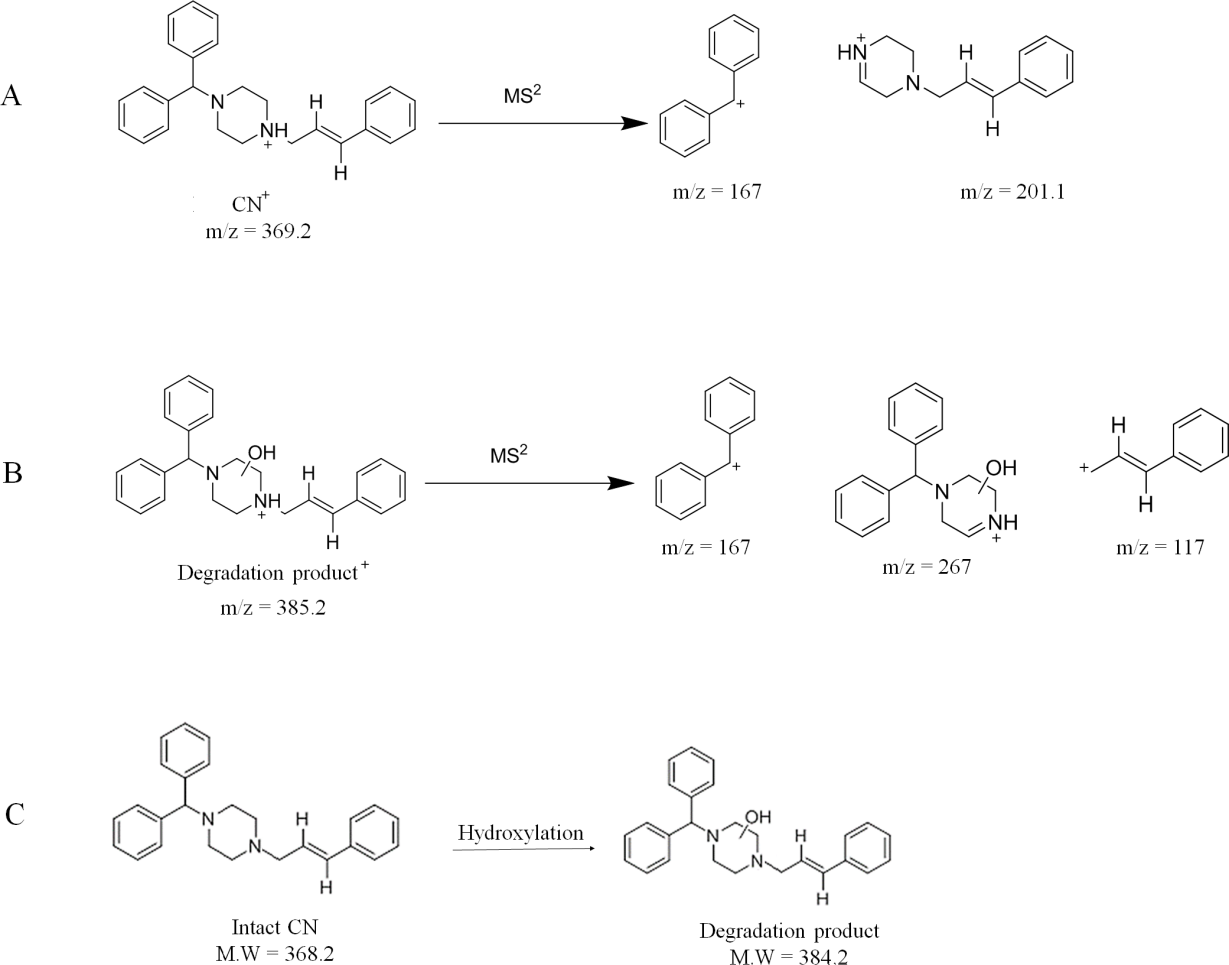
Schematic diagram of fragmentation pattern and degradation pathway of CN sample. (A) Fragmentation pattern for [CN]+ ion at m/z 369.2; (B) Fragmentation pattern for [degradation product]+ ion at m/z 385.2; (C) Proposed degradation pathway of CN within lipid-based formulation.

The UPLC/MS chromatogram of degraded CN-liquid SNEDDS sample showed the elution of CN and its main degradation product at ≈ 2.9 and 4.2 min, respectively (Fig 2A). The CN molecular ion [M + H]+ appeared at m/z 369.2 while the degradation product [M + H]+ appeared at m/z 385.2. The difference between them = 16 z/m which represents the introduction of one hydroxyl group to CN molecule. The MS^2^ mass spectrum for [CN]+ ion (m/z 369.2) gave two major product ions at m/z 167 and 201.1 (Fig 2B, Fig 3A and S1 Supporting Information) while the MS^2^ mass spectrum for the degradation product (m/z 385.2) gave three major product ions at m/z 117.1, 167.1 and 267.4 (Fig 2C, Fig 3B and S2 Supporting Information). These data suggests that the hydroxyl group was introduced to one of the piperazine carbons (Fig 3B and 3C) [38]. Accordingly, CN degradation, within lipid-based formulation, involved a hydroxylation reaction which could be catalyzed by the presence of free fatty acid in the SNEDDS formulation. Similar hydrolytic degradation have been reported with other drugs such as cefpodoxime proxetil and simvastatin in liquid SNEDDS [39]. CN was also reported to undergo extensive hydrolytic degradation within aqueous systems [4, 40].

### Pellet size and size distribution

**Fig 4:**
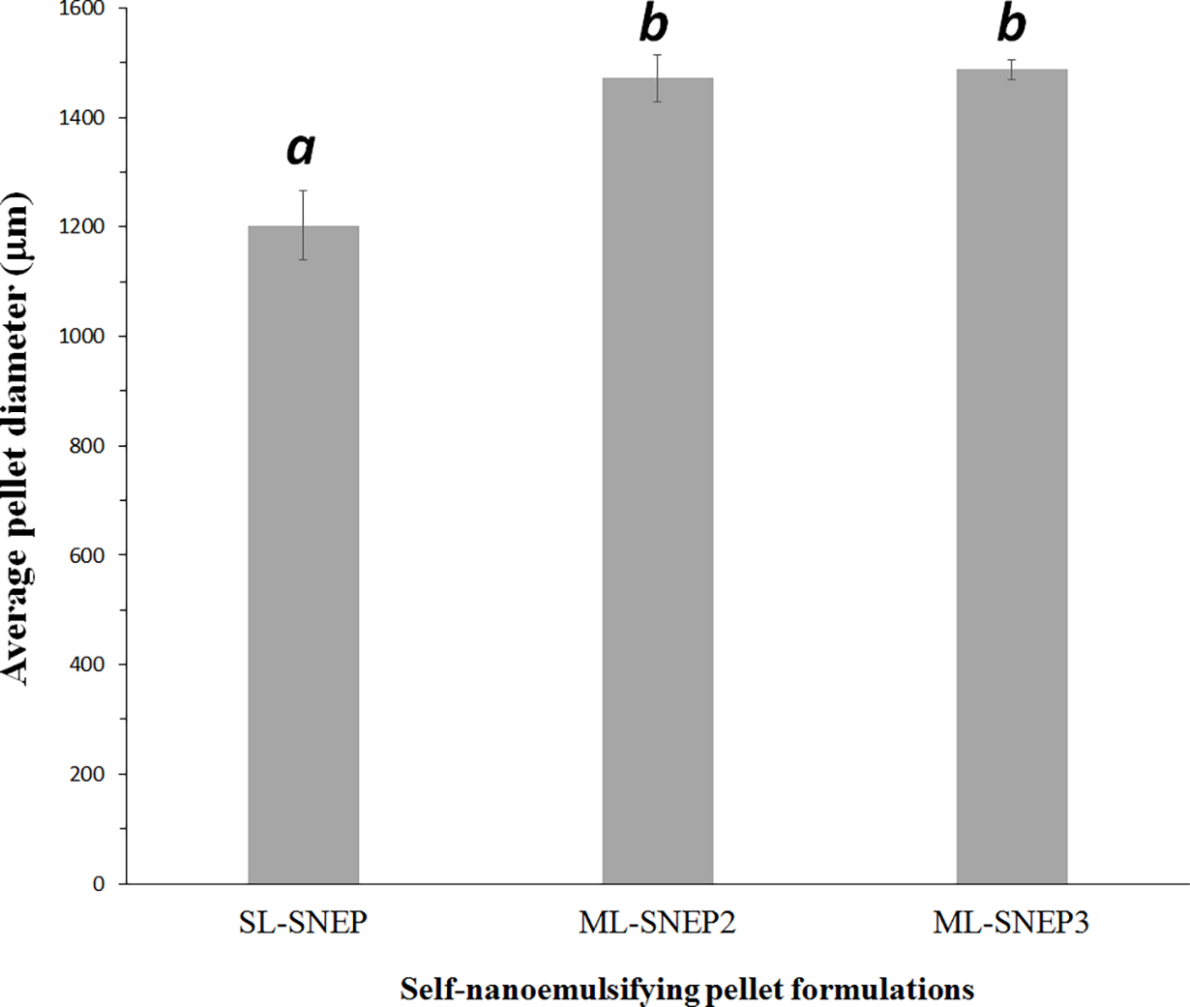
Average pellet diameter of CN self-nanoemulsifying pellet formulations. Different letters above the bars indicate significant difference (p<0.05) between the values and vice versa.

SL-SNEP showed significantly (*p< 0*.*05*) lower pellet diameter compared to ML-SNEP2 and ML-SNEP3 (Fig 4). Similarly, the sieve analysis study showed that (> 92%) of SL_SNEP were in the size range 1000–1250 μm. Whereas (> 97%) of ML-SNEP2 and ML-SNEP3 were in the size range 1250–1600 μm (Table 6). These results can reflect the significant increase in pellet size upon switching from single layer to multi-layer systems.

**Table 6:** Pellet size and distribution of single and multi-layer pellets.

Sieve Size (μm)	SL-SNEP*	ML-SNEP2*	ML-SNEP3*
600–800	0.0	0.0	0.0
800–1000	0.1	<0.4	<0.6
1000–1250	92.4	<0.4	<0.6
1250–1600	5.5	97.8	98.5
1600–1700	0.2	0.7	<0.6
1700–2000	0.2	1.0	<0.6
>2000	1.5	<0.4	<0.6

*Data are expressed as mean of three replicates showing the proportion of pellets present within each size range.

### Chemical stability studies

#### Accelerated stability study

**Fig 5:**
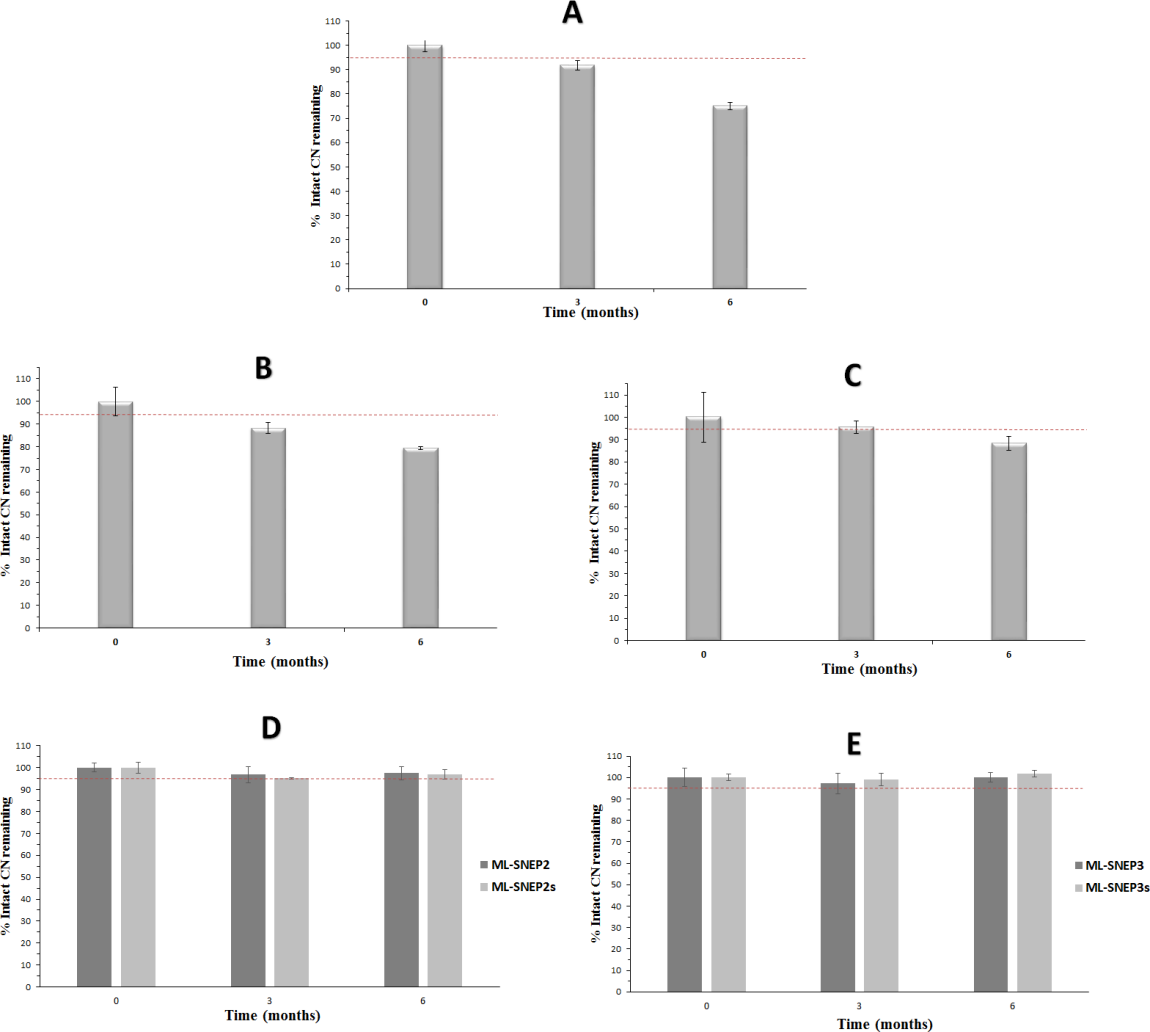
Accelerated chemical stability data of CN formulations. (A) liquid SNEDDS; (B) SL-SNEP; (C) ML-SNEP1; (D) ML-SNEP2 and ML-SNEP2s; (E) ML-SNEP3 and ML-SNEP3s. The formulations were stored for a minimum of 6 months at 40°C ± 2°C, 75% ± 5% RH. Data are expressed as mean±S.D, n = 6–12.

At the accelerated storage conditions, CN showed significant degradation (p < 0.05) in liquid SNEDDS at 3 and 6 months samples (Fig 5A). At 6 months sample, the intact CN amount decreased to 75% of the initial value. These results are in agreement with previously published work [4, 5] where CN experienced significant degradation within liquid SNEDDS and particularly the oil components.

Solidification into SL-SNEP substantially decreased CN degradation within the formulations. However, CN degradation was still significant at 3 and 6 months samples (Fig 5B). At 6 months sample, the intact CN amount decreased to 79% of the initial value. The direct contact between drug and SNEDDS excipients led to significant drug degradation which continued to occur in solid state but at slower rate relative to liquid state [25].

Solidification into ML-SNEP resulted into significant enhancement of CN stability within the formulation. ML-SNEP1 could maintain 86% of the initial CN value after 6 months of storage (Fig 5C). In fact, ML-SNEP1 showed significant enhancement of CN stability (compared to liquid SNEDDS and SL-SNEP). However, significant (p < 0.05) CN degradation was still observed at the 6 months sample. The enhanced stability of ML-SNEP1 is owing to the complete isolation of CN layer from the SNEDDS layer, via a protective layer [24]. This innovative design could substantially eradicate CN degradation within SENDDS. However it seems that CN was still susceptible to hydrolysis degradation due to the lack of moisture sealing layer in ML-SNEP1. This suggestion was confirmed by the subsequent stability findings of moisture sealed pellets.

Interestingly, the moisture sealed ML-SNEP (ML-SNEP2 and ML-SNEP3) showed significant enhancement of the CN stability, compared to all the previous formulations. ML-SNEP2 and ML-SNEP3 could maintain a minimum of 96% and 97% of initial CN value, respectively (Fig 5D and 5E). These outstanding results are owing to the complete isolation between CN and SNEDDS layer as well as incorporation of the moisture-sealing layer on top of the CN layer. The moisture sealing layer could provide effective protection against water vapor. Accordingly, CN was dually protected against degradation by SNEDDS components as well as water vapor. Hence, the degradation problem could be eradicated completely and both ML-SNEP2 and ML-SNEP3 could fulfill the ICH accelerated stability requirements maintaining ≥ 95% intact CN initial values [34].

#### Influence of anti-adherent layer

The incorporation of silicon dioxide onto ML-SNEP had no significant influence on the CN stability at accelerated conditions. There was no significant difference between ML-SNEP2 and its counterpart formulation comprising silicon dioxide (ML-SNEP2s) within accelerated stability data (Fig 5D). Similar findings were observed between ML-SNEP3 and ML-SNEP3s (Fig 5E). Generally, Both ML-SNEP2s and ML-SNEP3s maintained ≥ 95% of intact CN initial values.

#### Intermediate stability study

According to ICH guidelines, If “significant change” occurs at the accelerated storage condition, additional testing at the intermediate storage condition should be conducted [34].

**Fig 6:**
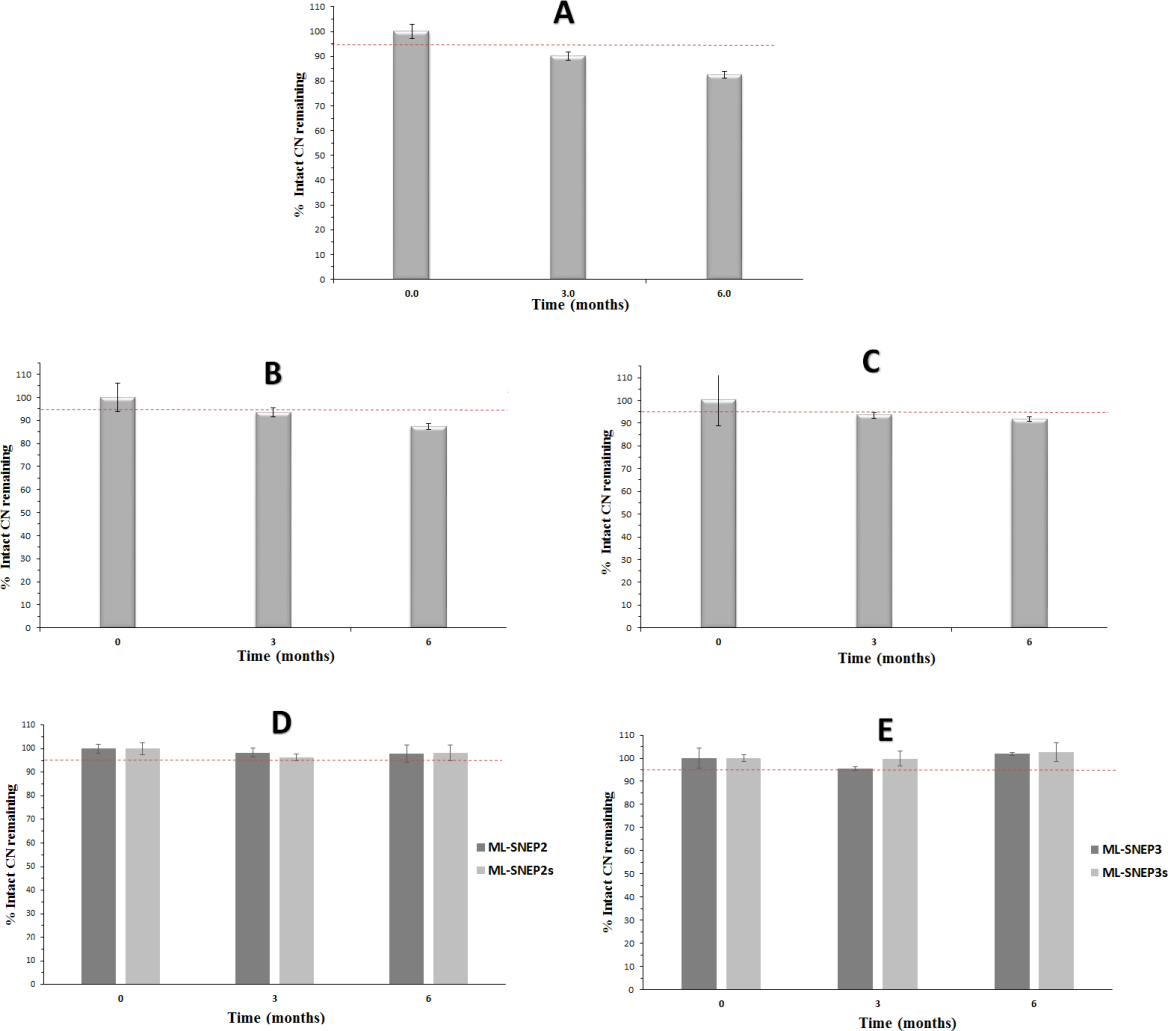
Intermediate chemical stability data of CN formulations. (A) liquid SNEDDS; (B) SL-SNEP; (C) ML-SNEP1; (D) ML-SNEP2 and ML-SNEP2s; (E) ML-SNEP3 and ML-SNEP3s. The formulations were stored for a minimum of 6 months at 30°C ± 2°C, 65% ± 5% RH. Data are expressed as mean±S.D, n = 6–12.

In fact, the results of intermediate stability study were strongly correlated with the accelerated stability findings. CN showed significant degradation (p < 0.05) in liquid SNEDDS at 3 and 6 months samples (Fig 6A). At 6 months sample, the intact CN amount decreased to 83% of the initial value.

Solidification into SL-SNEP substantially decreased CN degradation rate within the formulations. However, CN degradation was still undergoing significantly (p < 0.05) (Fig 6B). At 6 months sample, the intact CN amount decreased to 87% of the initial CN value.

Solidification into ML-SNEP resulted into significant enhancement of the CN stability within the formulation. At the end of the stability study, the ML-SNEP lacking moisture sealing (ML-SNEP1) could maintain > 91% of the intact CN initial value (Fig 6C). ML-SNEP1 showed significant enhancement of CN stability (compared to liquid SNEDDS and SL-SNEP). However, these pellets showed ≥ 5% change from the initial CN value and hence could not fulfill the ICH acceptance criteria [34].

Interestingly, the moisture sealed ML-SNEP (ML-SNEP2 and ML-SNEP3) showed significant enhancement of CN stability, compared to all the previous formulations. At the end of the study, ML-SNEP2 and ML-SNEP3 could maintain ≥ 95% of intact CN initial value (Fig 6D and 6E).

#### Influence of anti-adherent layer

Similar to their counterparts, both ML-SNEP2s and ML-SNEP3s maintained ≥ 95% of intact CN initial values, at the end of the study (Fig 6D and 6E).

#### Long-term stability study

**Fig 7:**
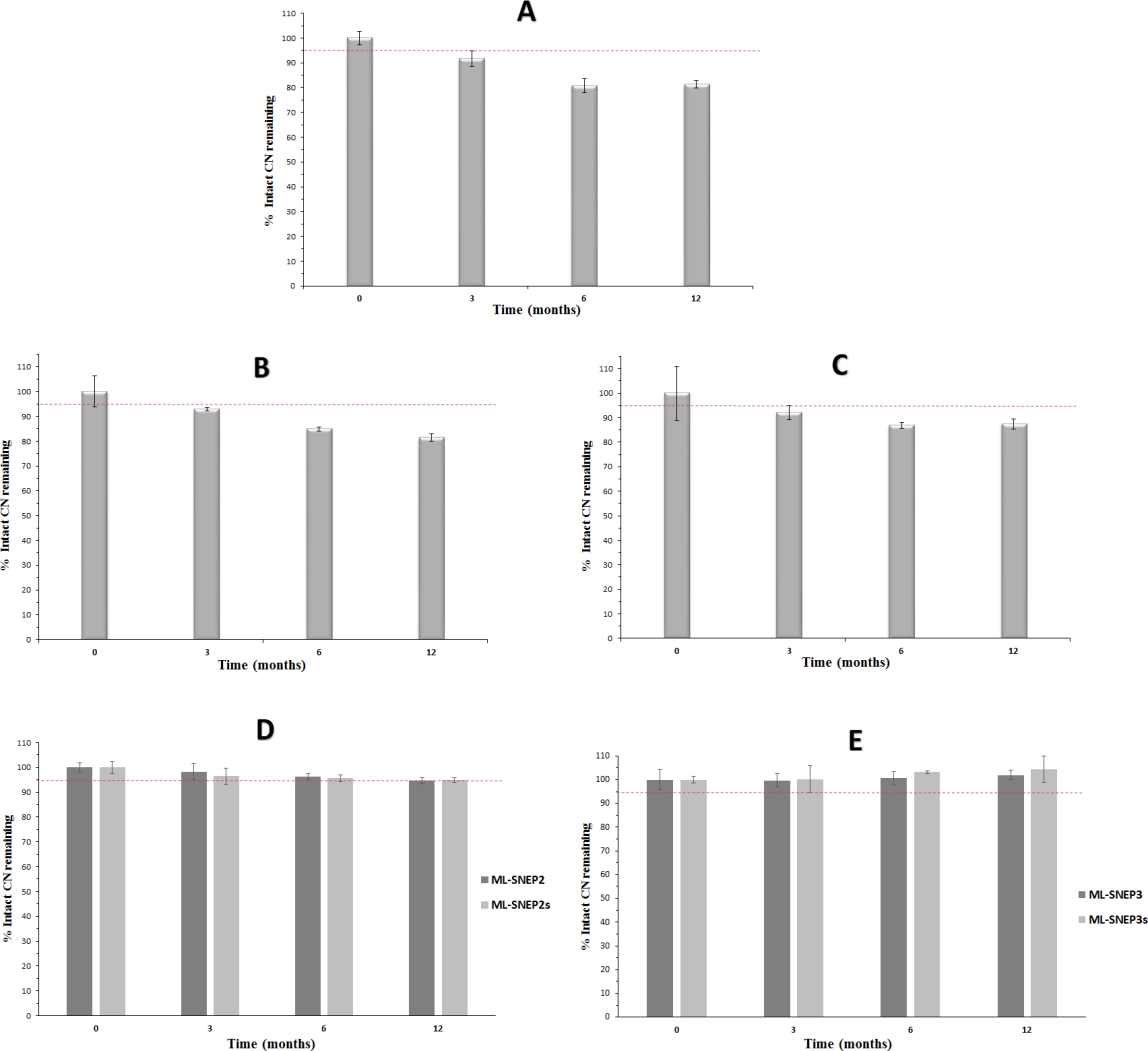
Long-term chemical stability data of CN formulations. (A) liquid SNEDDS; (B) SL-SNEP; (C) ML-SNEP1; (D) ML-SNEP2 and ML-SNEP2s; (E) ML-SNEP3 and ML-SNEP3s. The formulations were stored for a minimum of 12 months at 25°C ± 2°C, 60% ± 5% RH. Data are expressed as mean±S.D, n = 6–12.

The long-term stability investigation is the most important tool to accurately assess the stability pattern of the drug at labeled storage conditions. The stability data of CN at long-term storage conditions showed similar pattern to the corresponding accelerated and intermediate conditions. At the long-term storage conditions, CN showed significant degradation (p < 0.05) in liquid SNEDDS at all the time-points (Fig 7A). At 12 months sample, the intact CN amount decreased to 81% of the initial value.

SL-SNEP showed comparable results to liquid SNEDDS in the long-term storage conditions. SL-SNEP showed significant (p < 0.05) CN degradation at all the time-points (Fig 7B). At 12 months sample, the intact CN amount decreased to 81% of the initial CN value.

Solidification into ML-SNEP resulted into significant enhancement (p < 0.05) of CN stability within the formulation, compared to liquid SNEDDS and SL-SNEP. ML-SNEP1 could maintain 87% of the intact CN initial value, at the end of the study period (Fig 7C). In fact, ML-SNEP1 still showed significant (p < 0.05) CN degradation within the long-term storage conditions.

Interestingly, the moisture sealed ML-SNEP (ML-SNEP2 and ML-SNEP3) showed significant enhancement of the CN stability, compared to all the previous formulations. However, ML-SNEP2 showed significant (p < 0.05) CN degradation within the long-term storage conditions. ML-SNEP2 showed a slight decrease of intact CN below 95% of the initial value (Fig 7D). On the other hand, ML-SNEP3 showed significant enhancement of CN stability and could maintain > 97% of intact CN initial value (Fig 7E). Although ML-SNEP2 and ML-SNEP3 showed comparable stability findings in accelerated and intermediate studies, ML-SNEP3 showed significant (p < 0.05) enhancement of chemical CN stability compared to ML-SNEP2, particularly at long-term storage conditions. This could be explained by the fact that ML-SNEP2 comprises HPMC E3 while ML-SNEP3 comprises Kollicoat Smartseal 30D, as moisture sealing polymers. HPMC E3 is a water-soluble hygroscopic polymer, therefore, it is not presenting sufficient protection of CN against water vapor. Alternatively, Kollicoat Smartseal 30D is soluble in acidic pH and insoluble in neutral or basic pH, hence it is highly impermeable to water vapor [41]. Accordingly, the superiority of ML-SNEP3 could be explained by the complete insensitivity of Kollicoat Smartseal 30D to water vapor leading to effective moisture protection of the coated pellets.

#### Influence of anti-adherent layer

Similar to their counterparts, ML-SNEP2s showed significant (p < 0.05) CN degradation within the long-term storage conditions (Fig 7D). Intact CN decreased to < 95% of its initial values. However, ML-SNEP3s showed enhanced CN stability and maintained ≥ 95% of intact CN initial values all over the study period (Fig 7E).

#### Assessment of the chemical stability findings

The chemical stability data revealed that CN was significantly degraded in liquid SNEDDS, SL-SNEP and ML-SNEP1 at all the storage conditions. Furthermore, CN was significantly degraded in ML-SNEP2 at long-term storage conditions.

Liquid SNEDDS presented the highest CN degradation rate. This is obviously owing to the liquid nature of the formulation and the direct contact of the drug with the SNEDDS excipients. This led to hydroxlyation of CN molecule due to the presence of free fatty acid within the formulation (Fig 3C).

Solidification into SL-SNEP decreased the CN degradation but could not stop it completely because the drug was still in direct contact with the solidified SNEDDS excipients (Fig 1A) [25]. This direct contact would imply the incidence of the hydrolytic degradation but at slower rates compared to the liquid counterpart formulation. Solidification into ML-SNEP1 led to further decrease in CN degradation rate which is owing to the complete isolation between CN layer and SNEDDS layer, via a protective layer (Fig 1B). However, CN was still susceptible to hydrolysis degradation by atmospheric moisture due to the lack of moisture sealing layer in ML-SNEP1. This hypothesis was confirmed by the significant enhancement of CN stability upon adding the moisture-sealing layer. The moisture sealed ML-SNEP (ML-SNEP2 and ML-SNEP3) showed significant enhancement of CN stability, compared to all the previous formulations (Fig 1C). This is owing to the complete isolation between CN and SNEDDS layer as well as incorporation of the moisture-sealing layer on top of the CN layer. Due to the highly hydrophilic nature of HPMC E3, ML-SNEP2 could not provide sufficient protection against atmospheric moisture especially at long-term storage conditions. While, ML-SNEP3 comprised the water-insoluble polymer “Kollicoat Smartseal 30D” that provided optimum protection of CN against moisture. Therefore, ML-SNEP3 showed the best CN chemical stability profile. At the end of the study, ML-SNEP3 maintained ≥ 95% of intact CN within all the storage conditions. In conclusion, the CN degradation was in the following order: liquid SNEDDS > SL-SNEP > ML-SNEP1 > ML-SNEP2 > ML-SNEP3, with significant (p < 0.05) difference between each formulation.

### *In-vitro* dissolution studies

#### Mechanism of CN dissolution from the pellets

**Fig 8:**
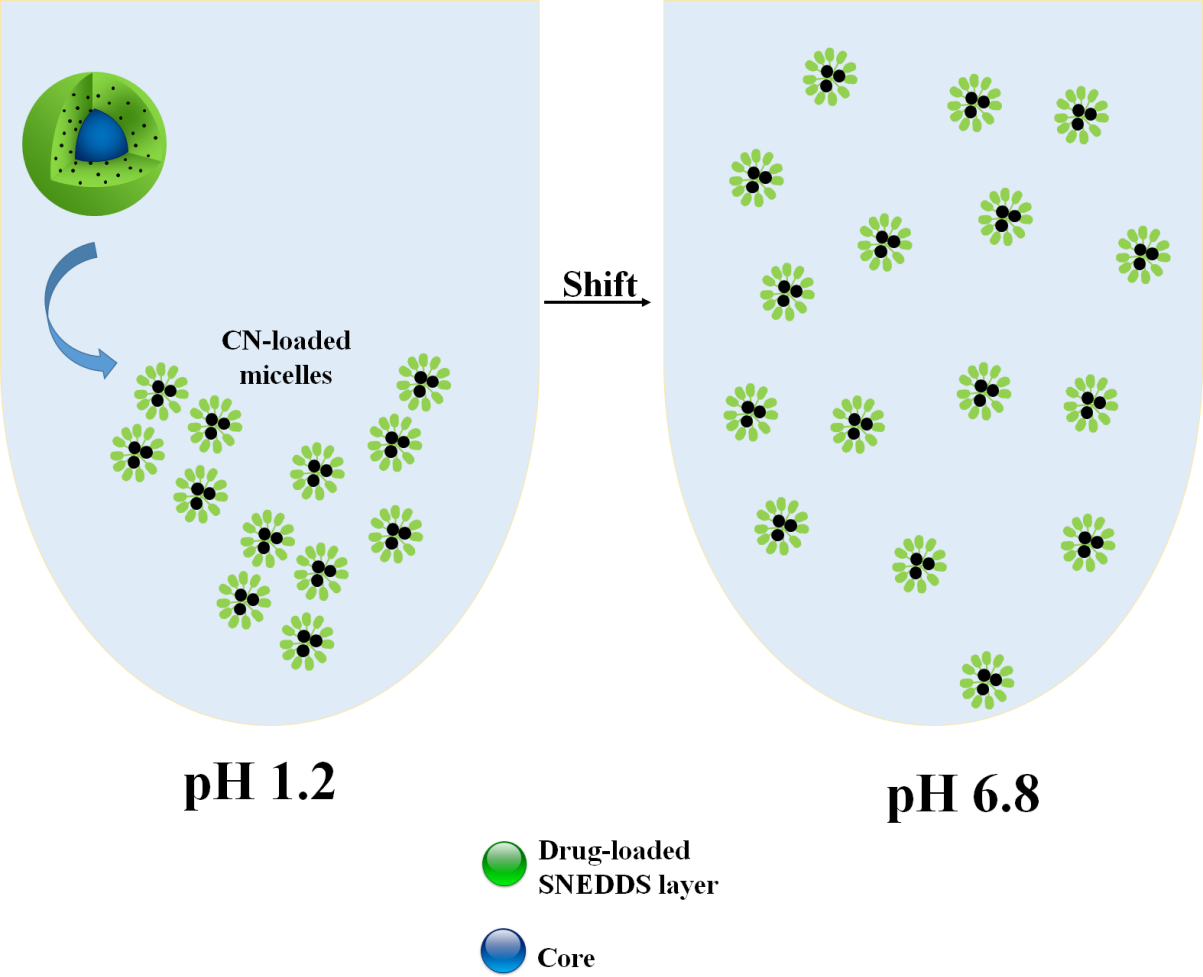
Schematic diagram of CN dissolution from SL-SNEP. The black spheroids represent CN particles.

**Fig 9:**
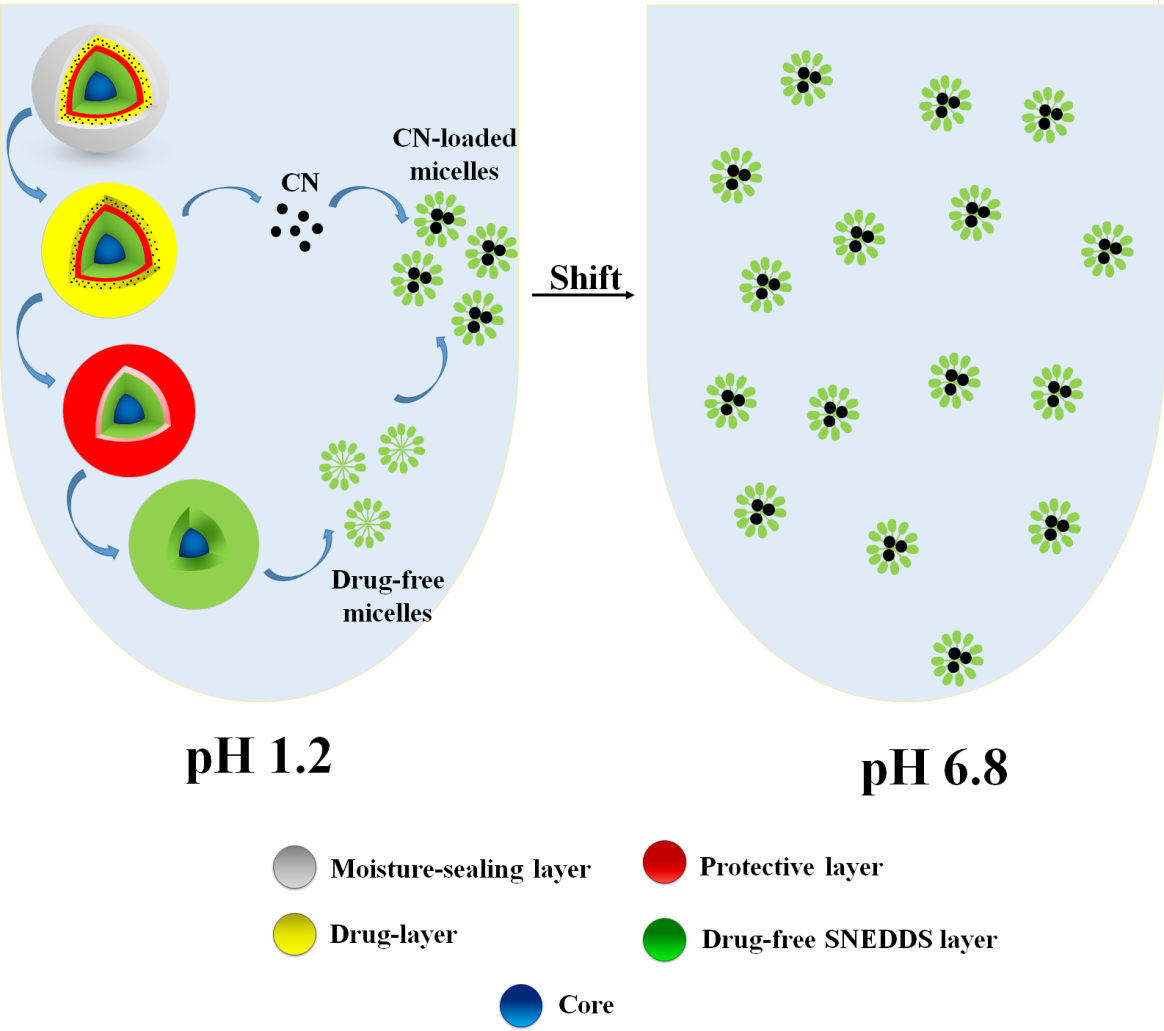
Schematic diagram of CN dissolution from ML-SNEP3. The black spheroids represent CN particles.

CN is a weak base that shows higher solubility at acidic pH and extensive precipitation upon shifting to higher pH [6]. This problem could be efficiently solved by using SNEDDS technology which enhanced drug dissolution at pH 1.2 and resisted extensive precipitation upon shifting to pH 6.8. SL-SNEP involved the CN-loaded SNEDDS in a single layer. Therefore, upon aqueous dispersion, the drug-loaded SNEDDS would spontaneously self-emulsify into CN-loaded micelles leading to enhanced CN solubilization at pH 1.2 and upon shifting to pH 6.8 (Fig 8). On the hand, ML-SNEP3 involved the water-insoluble polymer “Kollicoat Smartseal 3D” as the outer layer. This polymer is insoluble in neutral and basic media while it is soluble in acidic media (pH ≤ 5.5). Therefore, the dissolution of ML-SNEP3 at pH 1.2 was initiated by the erosion of the moisture sealing layer followed by CN release from the drug layer into the acidic media (Fig 9). Subsequently, the water-soluble “protective layer” would be eroded. Finally, the drug-free SNEDDS layer would spontaneously self-emulsify into drug-free micelles which would tend to encapsulate the released CN particles to form CN-loaded micelles. These micelles would result in enhanced CN solubilization at pH 1.2 and even after shifting to pH 6.8.

#### Accelerated stability study

**Fig 10:**
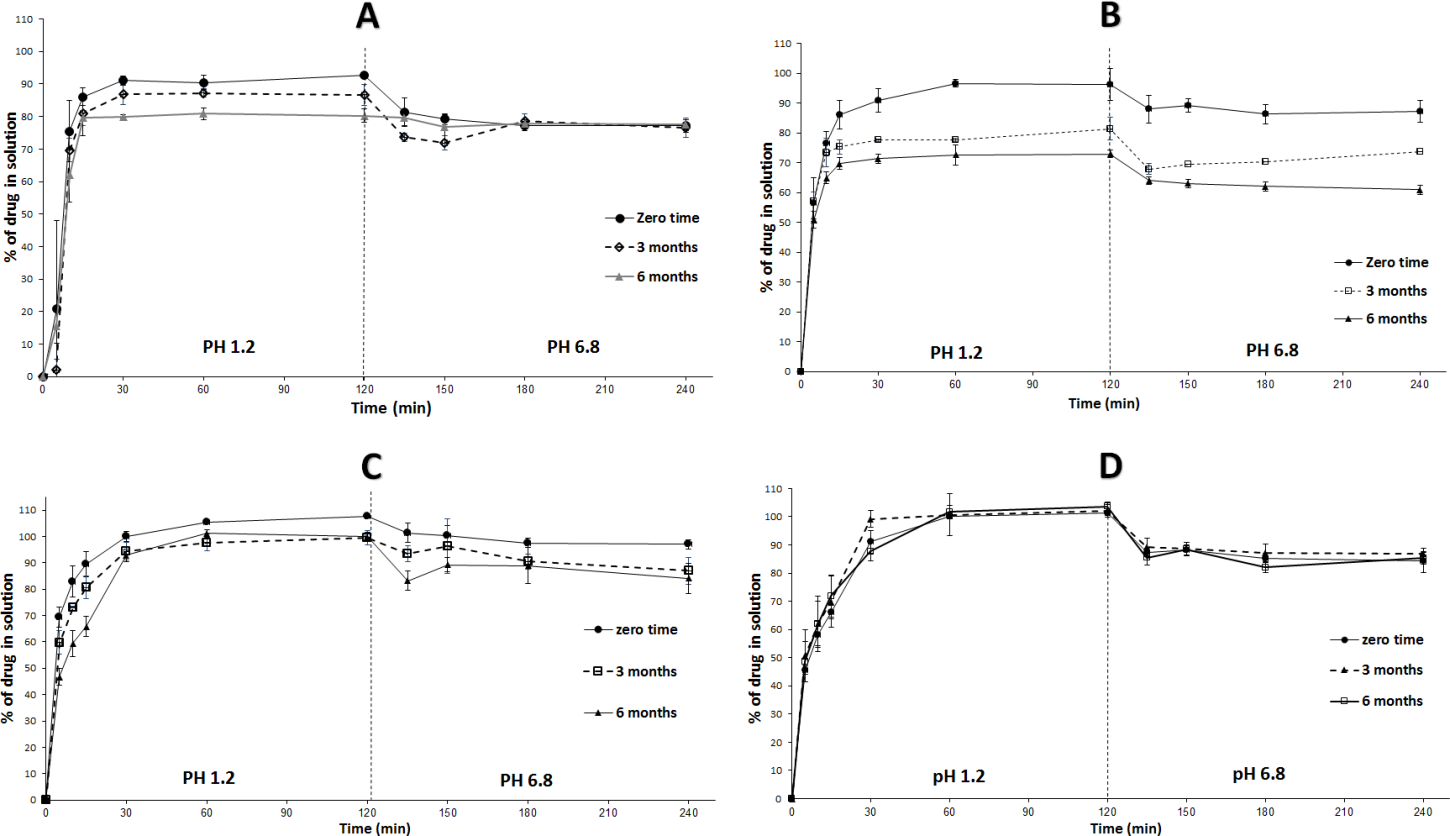
*In-vitro* dissolution of CN formulations at accelerated storage conditions. (A) Liquid SNEDDS; (B) SL-SNEP; (C) ML-SNEP2; (D) ML-SNEP3. The formulations were stored for a minimum of 6 months at 40°C ± 2°C, 75% ± 5% RH. Data are expressed as mean ± SD, n = 3–6.

The findings of *in-vitro* dissolution studies were strongly correlating with the chemical stability findings. In general, the formulations that showed significant CN degradation presented significant decrease in DE and vice versa. The *in-vitro* dissolution studies of liquid SNEDDS and SL-SNEP revealed significant (p < 0.05) decrease in DE, at 3 and 6 months (Fig 10A and 10B). However, both liquid SNEDDS and SL-SNEP didn't show aggressive precipitation upon shifting to pH 6.8 which ensures that all the formulations retained their emulsification efficiency and general dissolution pattern. These findings are strongly correlating with the corresponding chemical stability findings (Fig 5A and 5B). The observed drop in DE is obviously owing to the decrease of intact CN remaining in formulation.

On the other hand, ML-SNEP1 showed significant pellet adherence upon storage as illustrated in the next “physical appearance” section. Therefore, it was not feasible to undergo *in-vitro* dissolution testing for the stored ML-SNEP1.

Compared to SL-SNEP, ML-SNEP2 showed substantial enhancement of CN dissolution from stored pellets. However, ML-SNEP2 still showed significant (p < 0.05) decrease in DE at 3 and 6 months samples (Fig 10C). Interestingly, ML-SNEP3 showed no significant decrease in DE at 3 and 6 months samples and all the dissolution profiles were nearly superimposed (Fig 10D). These results are in good correlation with the corresponding chemical stability findings where ML-SNEP3 maintained > 97% of intact CN initial value (Fig 5E).

#### Long-term stability study

**Fig 11:**
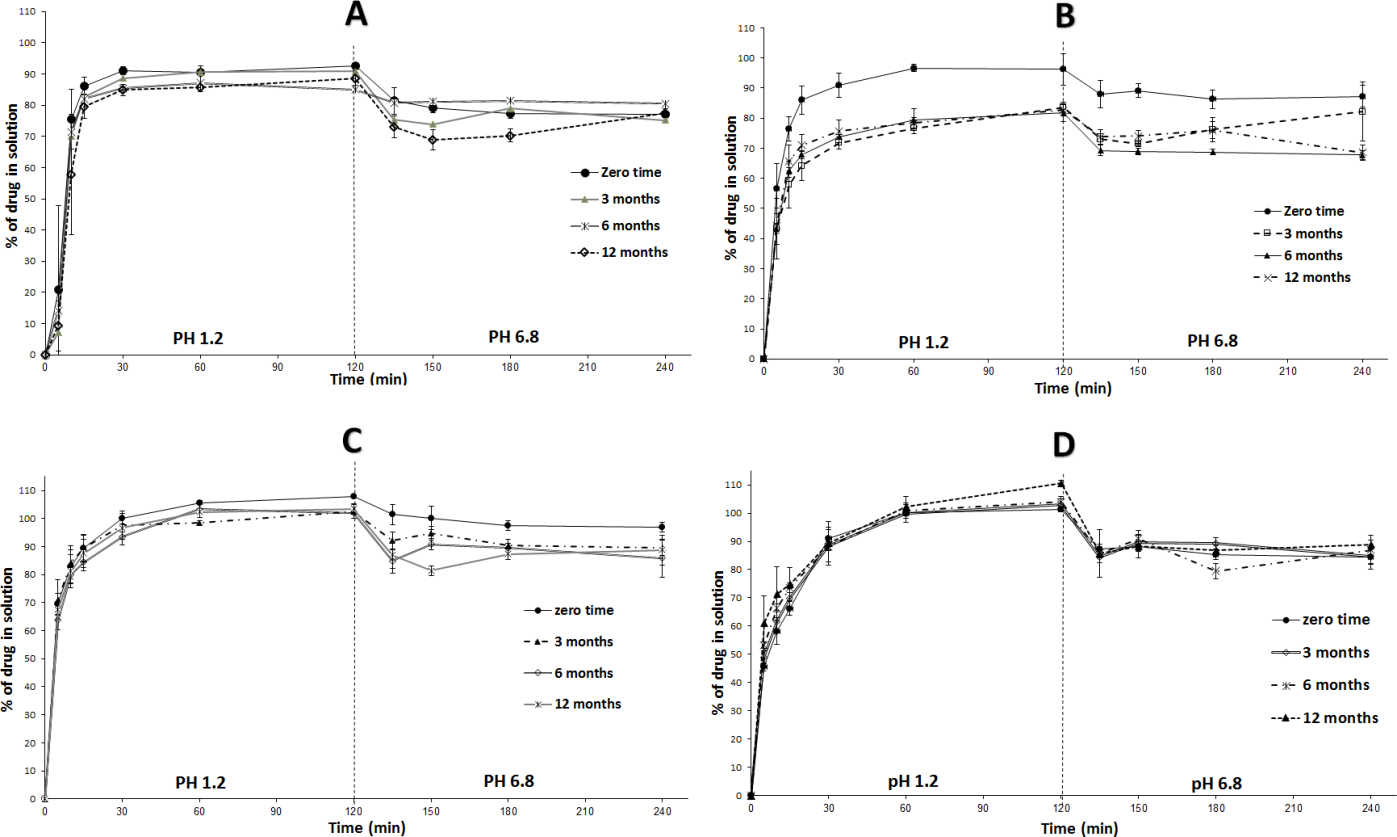
*In-vitro* dissolution of CN formulations at long-term storage conditions. (A) Liquid SNEDDS; (B) SL-SNEP; (C) ML-SNEP2; (D) ML-SNEP3. The formulations were stored for a minimum of 12 months at 25°C ± 2°C, 60% ± 5% RH. Data are expressed as mean ± SD, n = 3–6.

The long-term stability findings were in good correlation with the corresponding accelerated stability results. Liquid SNEDDS, SL-SNEP and ML-SNEP2 showed significant (p < 0.05) decrease in DE at the end of the long-term study (Fig 11A–11C). These findings are also strongly correlating with the corresponding chemical stability findings (Fig 7A, 7B and 7D). The observed drop in DE is mostly owing to the decrease of intact CN remaining in formulation. On the other hand, ML-SNEP3 showed no significant drop of DE all over the study period (Fig 11D).

#### Influence of anti-adherent layer

**Fig 12:**
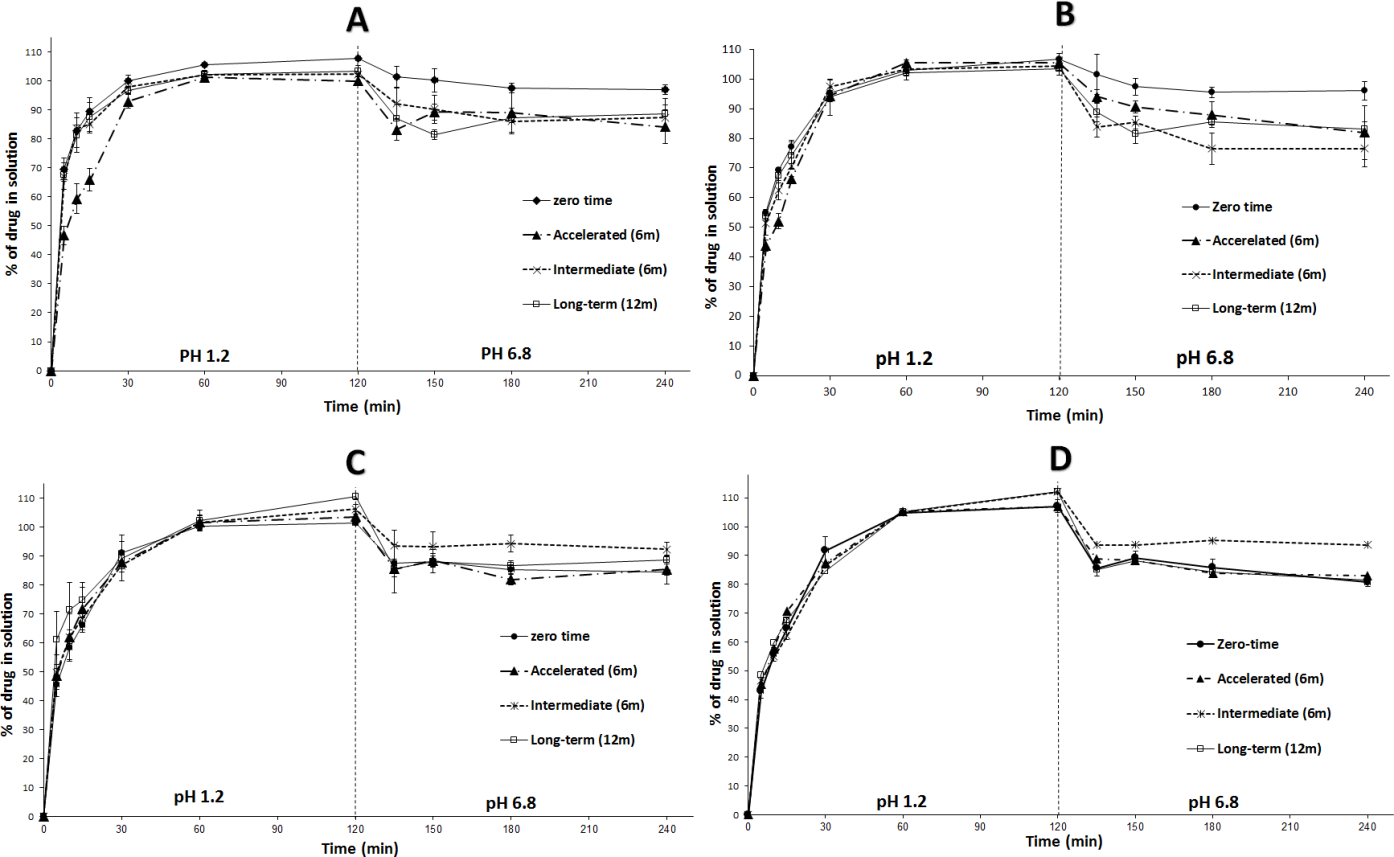
Influence of anti-adherent layer on the *in-vitro* dissolution of ML-SNEP at different storage conditions. (A) ML-SNEP2; (B) ML-SNEP2s; (C) ML-SNEP3; (D) ML-SNEP3s. Accelerated and intermediate data involved storage for a minimum of 6 months and long-term data involved storage for a minimum of 12 months at relevant conditions. Data are expressed as mean ± SD, n = 3–6. ML-SNEP denotes: multi-layer self-nanoemulsifying pellets.

The *in-vitro* dissolution of ML-SNEP2s and ML-SNEP3s (upon storage) was in good correlation with their counterparts that lack silicon dioxide; ML-SNEP2 and ML-SNEP3, respectively. At the end of all stability studies, both ML-SNEP2 and ML-SNEP2s showed significant (p < 0.05) decrease in DE (Fig 12A and 12B). In contrast, both ML-SNEP3 and ML-SNEP3s showed no significant decrease in DE within all the storage conditions (Fig 12C and 12D). In general, both formulations comprising and lacking silicon dioxide showed correlated stability profiles. Hence, the addition of silicon dioxide had no considerable influence on formulation functionality upon storage.

#### Assessment of *in-vitro* dissolution findings

The previous dissolution findings revealed that liquid SNEDDS, SL-SNEP and ML-SNEP2 showed significant (p < 0.05) decrease of DE within all the tested conditions. Regarding stability aspects, ML-SNEP3 showed the best *in-vitro* dissolution profile since it did not show any significant decrease in DE all over the storage conditions. These data are strongly correlating with the corresponding chemical stability findings. The enhanced stability profile of ML-SNEP3 is owing to the complete isolation between CN and SNEDDS layer as well as the effective moisture protection provided by Kollicoat Smartseal 30D. Accordingly, CN was dually protected against degradation by SNEDDS components and water vapor.

### Physical appearance

**Fig 13:**
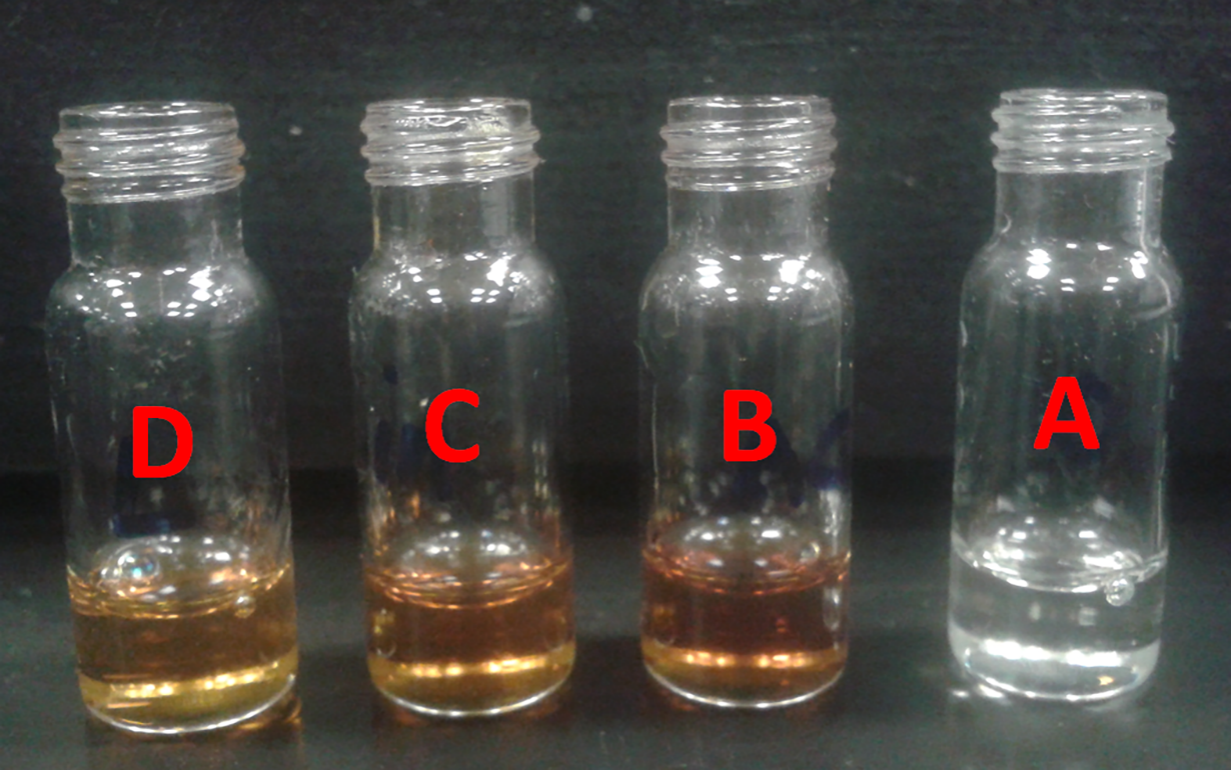
Digital images of CN liquid SNEDDS at different storage conditions. (A) Freshly prepared liquid SNEDDS; (B) Liquid SNEDDS after a minimum of 6 months of accelerated storage conditions; (C) Liquid SNEDDS after a minimum of 6 months of intermediate storage conditions; (D) Liquid SNEDDS after a minimum of 12 months of long-term storage conditions.

The physical appearance investigation revealed that liquid SNEDDS experienced sharp discoloration at accelerated, intermediate and long-term storage conditions (Table 7, Fig 13). The formulation appearance changed from transparent yellow to brown and continued to go darker with time. This could be because liquid SNEDDS contain the unsaturated long chain fatty acid (OL). The susceptibility of the lipid to oxidative rancidity depends upon its degree of unsaturation. Unsaturated fatty acids are more prone to oxidative rancidity than saturated ones [5]. In fact, this is one of the common drawbacks that limits the industrial utilization of liquid SNEDDS in oral drug delivery and could be solved by SNEDDS solidification.

**Table 7:** Physical appearance of CN formulations after a minimum of 6 months of accelerated, 6 months of intermediate and 12 months of long-term storage conditions.

Formulation	Physical appearance				Comments
	Zero time	Accelerated (6m)	Intermediate (6m)	Long term (12m)	
**Liquid SNEDDS**	Yellow	Brown	Brown	Brown	-
**SL-SNEP**	White	Yellowish white	Yellowish white	Yellowish white	• No pellet adherence
**ML-SNEP1**	White	Brown	Yellowish white	White	• Significant adherence at all conditions
**ML-SNEP2**	White	Brown	Yellowish white	Yellow	• No pellet adherence
**ML-SNEP2s**	White	Coffee	Yellowish white	Yellowish white	• No pellet adherence
**ML-SNEP3**	White	Brown	Yellowish white	Yellowish white	• Moderate adherence at accelerated
**ML-SNEP3s**	White	Coffee	Yellowish white	White	• No pellet adherence

**Fig 14:**
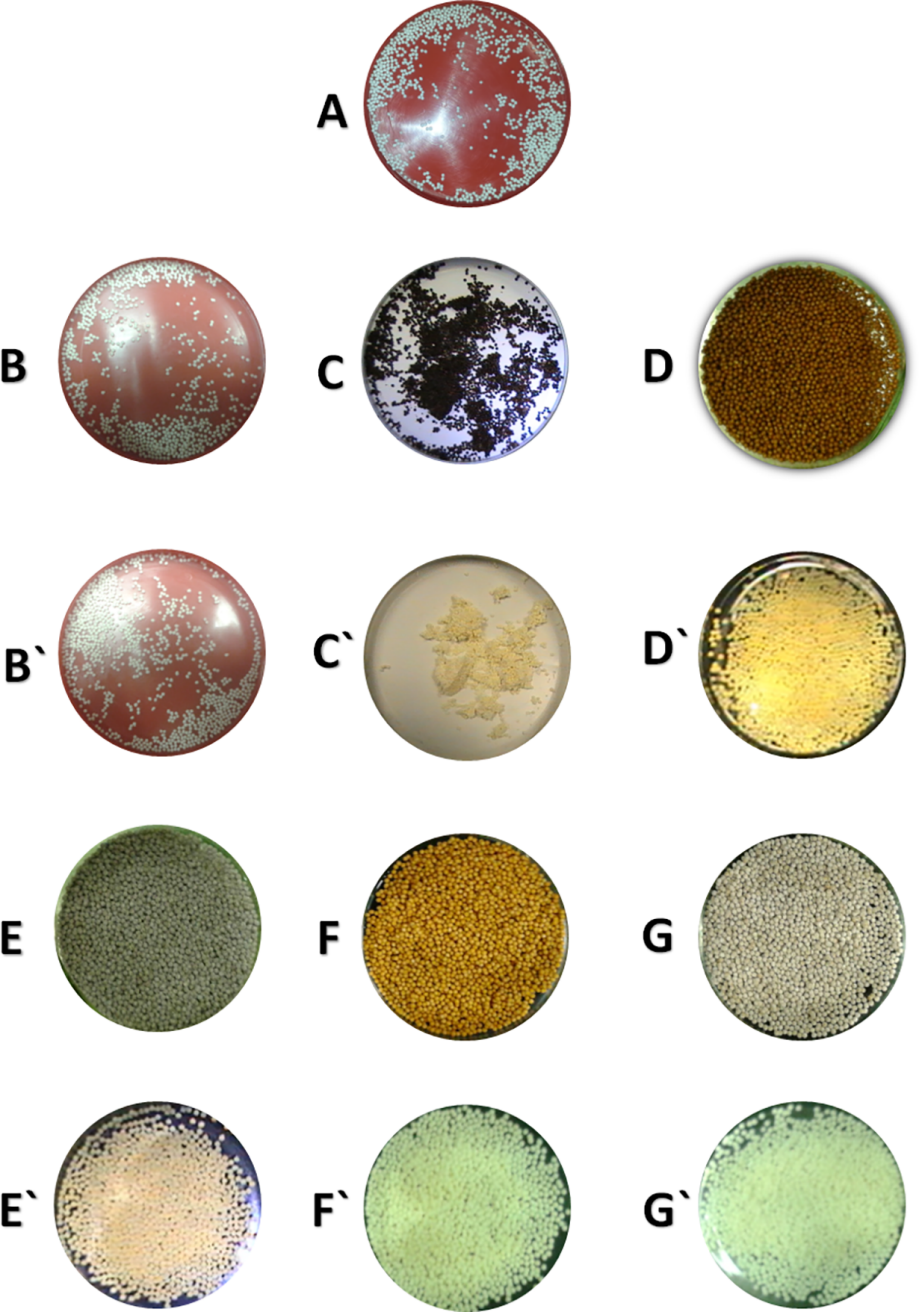
Digital images of CN self-nanoemulsifying pellets. (A) Freshly prepared representative pellets; (B) SL-SNEP; (C) ML-SNEP1; (D) ML-SNEP2; (E) ML-SNEP2s; (F) ML-SNEP3; (G) ML-SNEP3s after storage at accelerated conditions; (B`) SL-SNEP; (C`) ML-SNEP1; (D`) ML-SNEP2; (E`) ML-SNEP2s; (F`) ML-SNEP3; (G`) ML-SNEP3s after storage at long-term conditions.

On the other hand, SL-SNEP offer the advantage of enhanced physical stability. These pellets didn`t show any significant discoloration or pellet agglomeration. At the end of accelerated, intermediate and long-term studies, SL-SNEP remained free-flowing, well-separated with minor yellowish discoloration (Table 7, Fig 14B and 14B`).

Interestingly, ML-SNEP1 showed sharp discoloration at accelerated storage conditions (Table 7 and Fig 14C). At the end of the study, the pellet color changed from white into brown. Most importantly, ML-SNEP1 developed strong irreversible pellet adherence at early stages of the accelerated study (Table 7 and Fig 14C). At the intermediate and long-term storage conditions, ML-SNEP1 didn`t present significant discoloration. However, the pellets maintained the significant adherence at all the storage conditions (Table 7 and Fig 14C`). This finding is owing to the presence of PVP K30 as an outer layer within the pellets. Previous studies showed that the PVP coated pellets exhibited significant pellet agglomeration and moisture absorption, upon storage [42]. This finding would obviously lead to unsuccessful dissolution profile because the adhered pellets would fail to self-emulsify at acceptable time. Accordingly, it is mandatory to add a moisture-sealing layer on top of the PVP layer.

The moisture sealed ML-SNEP2 and ML-SNEP3 showed significant improvement of the latter adherence problem. ML-SNEP2 maintained its free-flowability within all the storage conditions. Significant discoloration was observed in the accelerated conditions while the intermediate and long-term studies revealed mild to moderate yellowish discoloration (Table 7, Fig 14D and 14D`). Similar findings were observed with ML-SNEP2s (Table 7, Fig 14E and 14E`). These pellets maintained its free-flowability within all the storage conditions. Yet significant discoloration was only observed at the accelerated storage conditions.

On the other hand, ML-SNEP3 developed mild adherence upon storage at accelerated conditions (Table 7 and Fig 14F). This could be due to the increased tackiness of Kollicoat SmartSeal 30D at high temperatures. However, this agglomeration was reversible and the pellets were easily separated from each other. Lower storage temperatures revealed no pellet adherence and the pellets maintained their free-flowability at the end of intermediate and long-term studies (Table 7 and Fig 14F`). The addition of silicon dioxide led to complete eradication of the agglomeration problem. Therefore, ML-SNEP3s showed no pellet adherence and maintained their free-flowability at all the storage conditions (Table 7, Fig 14G and 14G`). Regarding the pellet color, both ML-SNEP3 and ML-SNEP3s showed significant discoloration only at accelerated conditions (Table 7, Fig 14F and 14G).

### Overall assessment

Regarding the chemical stability study, the CN degradation was in the following order: liquid SNEDDS > SL-SNEP > ML-SNEP1 > ML-SNEP2 > ML-SNEP3, with significant (p < 0.05) difference between each formulation. ML-SNEP3 and its counterpart (ML-SNEP3s) showed the best CN chemical stability profile and maintained ≥ 95% of intact CN at the end-time of all the storage conditions.

With respect to *in-vitro* dissolution studies, liquid SNEDDS, SL-SNEP and ML-SNEP2 showed significant decrease of DE within all the tested conditions. However, these formulations maintained their general pattern with no aggressive precipitation after shifting to pH 6.8. Regarding the *in-vitro* dissolution studies, ML-SNEP3 showed the best stability profile since it showed no significant drop in DE within all the storage conditions.

Regarding the physical appearance, liquid SNEDDS experienced sharp discoloration at all the storage conditions. While SL-SNEP showed no significant change in physical appearance within all the storage conditions. Interestingly, ML-SNEP1 experienced significant pellet adherence at all the storage conditions. On the other hand, the moisture sealed ML-SNEP2 and ML-SNEP3 showed significant improvement of the latter adherence problem. These pellets suffered significant discoloration only at accelerated storage conditions.

The incorporation of silicon dioxide layer had no crucial influence on the chemical stability or *in-vitro* dissolution of ML-SNEP. However, it had an important role in inhibiting pellet agglomeration upon storage. Silicon dioxide was added as a top powder onto the pellets which led to adhesion of silicon dioxide particles to the pellet surfaces. This adhesion caused desirable roughness of the pellet surface and presented a physical barrier against dherence of the outer tacky layers of pellets (Fig 1D) [43]. Accordingly, silicon dioxide could effectively inhibit pellet agglomeration upon storage.

ML-SNEP3 showed no significant decrease in CN chemical assay or *in-vitro* dissolution at the end of all the storage conditions. Furthermore, these pellets maintained acceptable physical appearance upon storage. Therefore, ML-SNEP3 would be an efficient dosage form that combine both enhanced CN solubilization and stabilization.

## Conclusion

The poor aqueous solubility and chemical instability of CN make the formulation of CN quite challenging. Solidification of liquid SNEDDS offer strong option to enhance both CN aqueous solubility and stability. Multi-layering along with moisture sealing offer the best stabilization results. Both chemical and physical stability of CN formulations were significantly enhanced within ML-SNEP coated with Kollicoat Smartseal 30D. Acceptable chemical stability profile often led to acceptable dissolution results. Silicon dioxide played a vital role in inhibiting pellet agglomeration upon storage. Therefore, ML-SNEP coated with Kollicoat Smartseal and silicon dioxide would be an efficient dosage form that combine innovative CN solubilzation and stabilization. In future, the adopted study design could be generalized for weakly basic lipophilic drugs that suffer significant degradation within lipids.

## Supporting information

S1 Supporting Information UPLC/MS autotune report for m/z 369.18. (PDF)

S2 Supporting Information UPLC/MS autotune report for m/z 385.14. (PDF)
