# Supplementary material for: Stabilization benefits of single and multi-layer self-nanoemulsifying pellets: A poorly-water soluble model drug with hydrolytic susceptibility
Source: PLoS One. 2018 Jul 19;13(7):e0198469. doi: 10.1371/journal.pone.0198469 (PMC6053139; doi:10.1371/journal.pone.0198469)

AutoTune Report

This report displays the results of the MRM development carried out as part of an autotune.

Calibration method : C:\MassLynx\DEFAULT.PRO\AcquDB\calibration.cal

MSMS Tune method : C:\MassLynx\Cinnarizinne.pro\ACQUDB\CNN.ipr

Daughter search data file : C:\MassLynx\Cinnarizinne.PRO\data\Product.raw

Date: Generated on Wed 21 Mar 2018 at 13:44

Cone Optimize Chromatogram for m/z 369.18

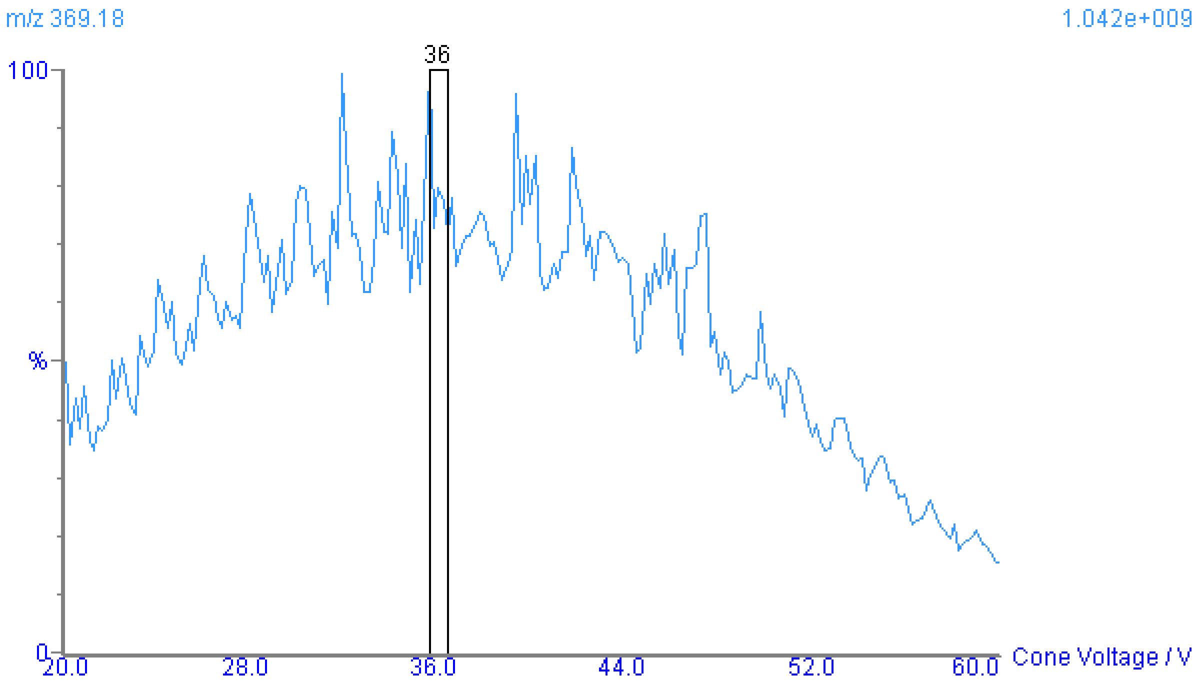

Cone Optimize Spectrum for m/z 369.18

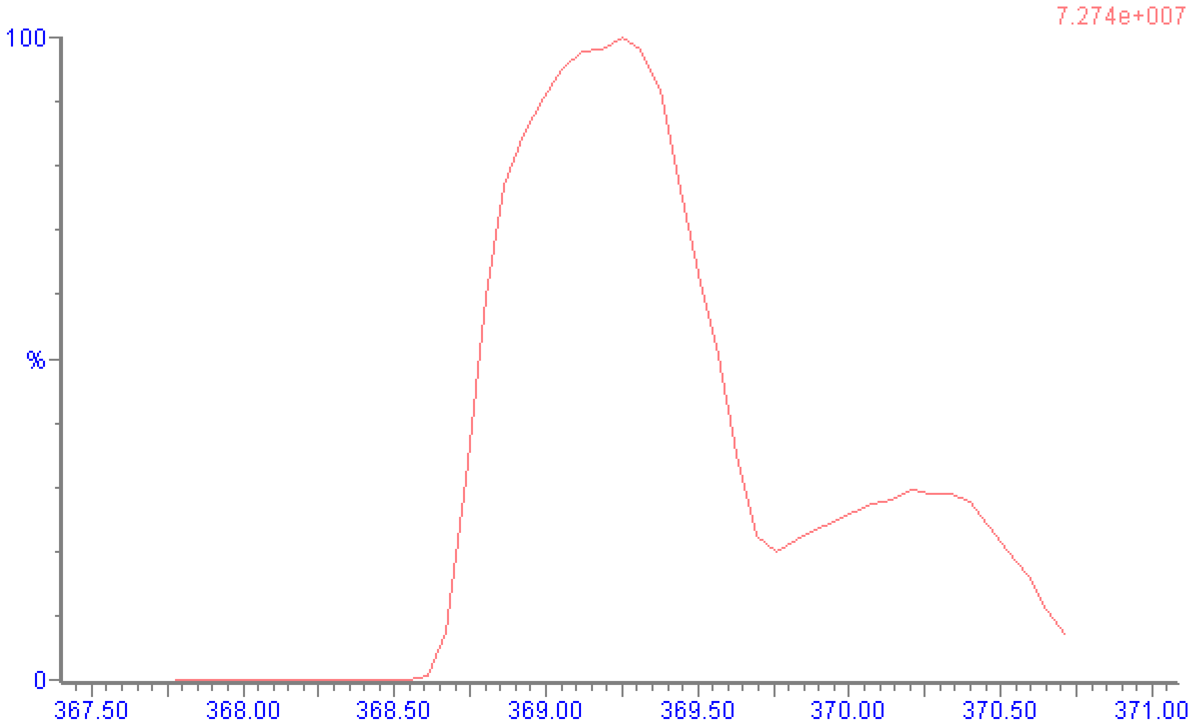

The MRM experiment developed: C:\MassLynx\Cinnarizinne.pro\ACQUDB\CNN Method.exp

Precursor Mass	Product Mass	Cone Voltage	Collision Energy
369.18	152.0	36.00	48.00
369.18	167.0	36.00	16.00
369.18	201.1	36.00	12.00

Collision Energy Optimize Chromatogram for m/z 369.18 -> 152.0

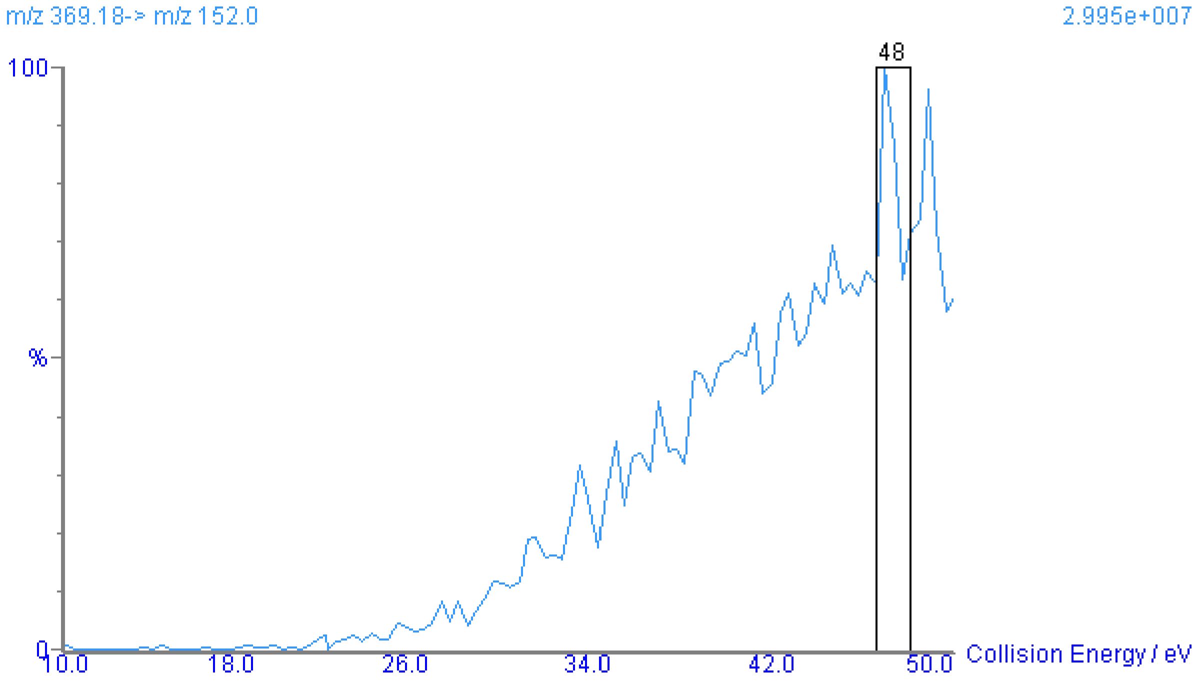

Collision Energy Optimize Spectrum for m/z 369.18 -> 152.0

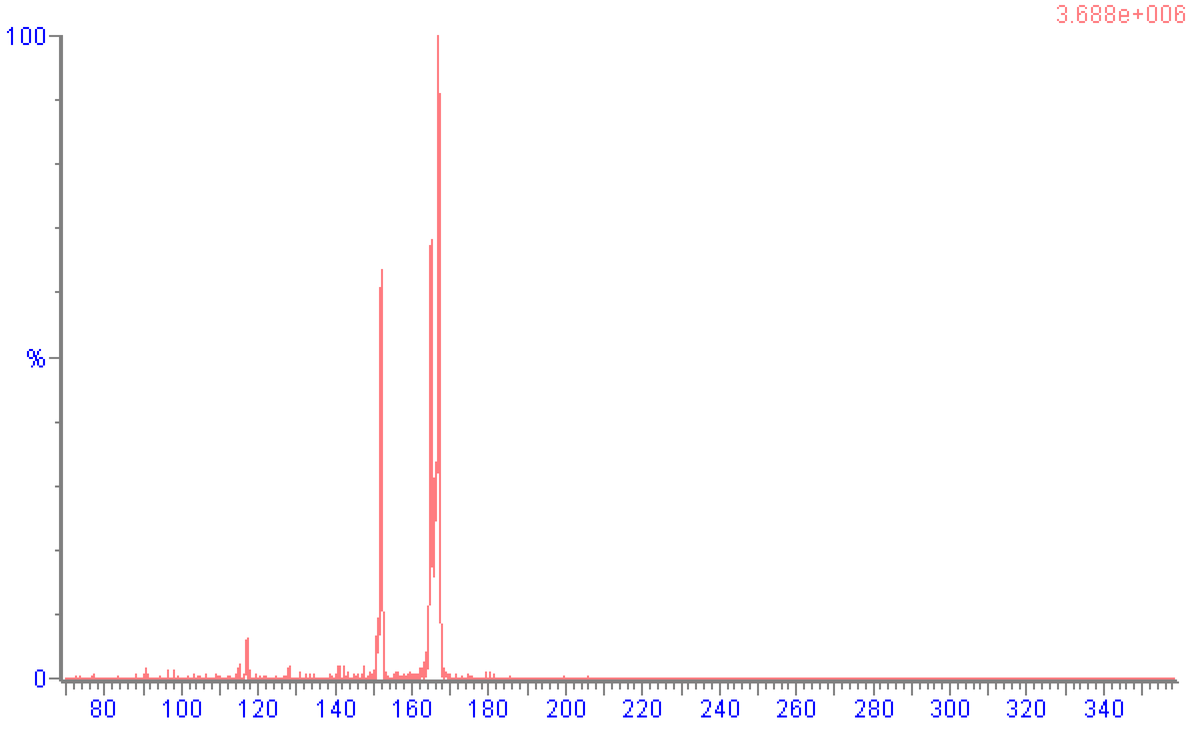

Collision Energy Optimize Chromatogram for m/z 369.18 -> 167.0

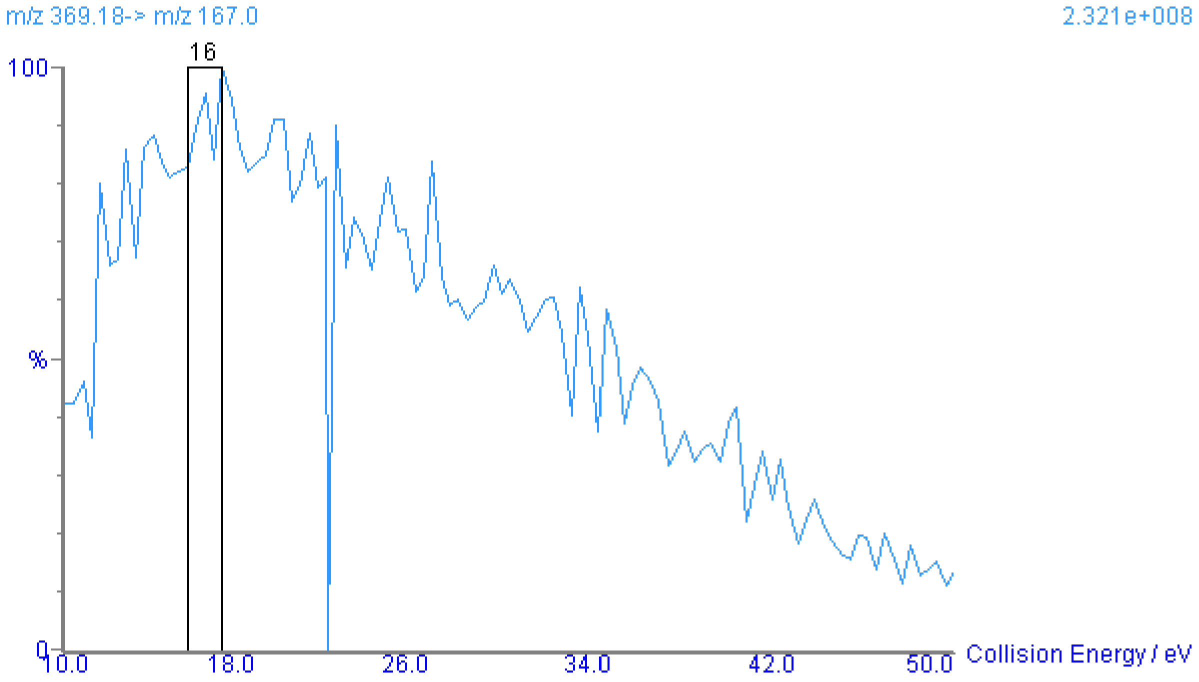

Collision Energy Optimize Spectrum for m/z 369.18 -> 167.0

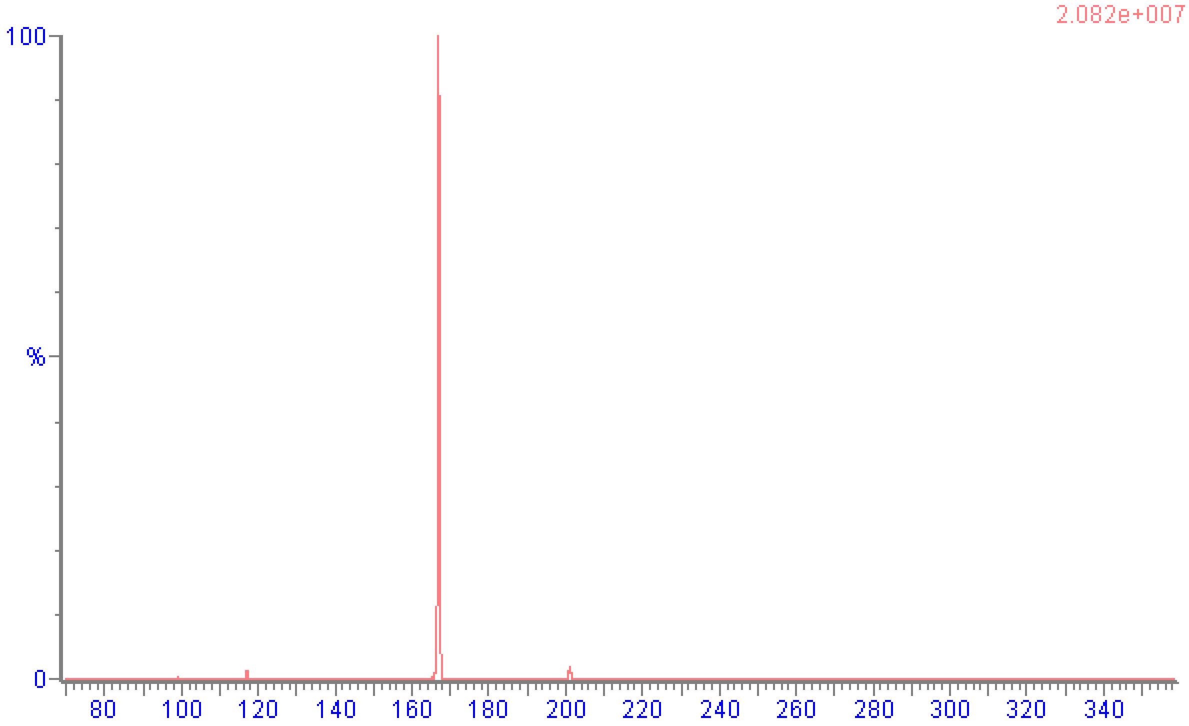

Collision Energy Optimize Chromatogram for m/z 369.18 -> 201.1

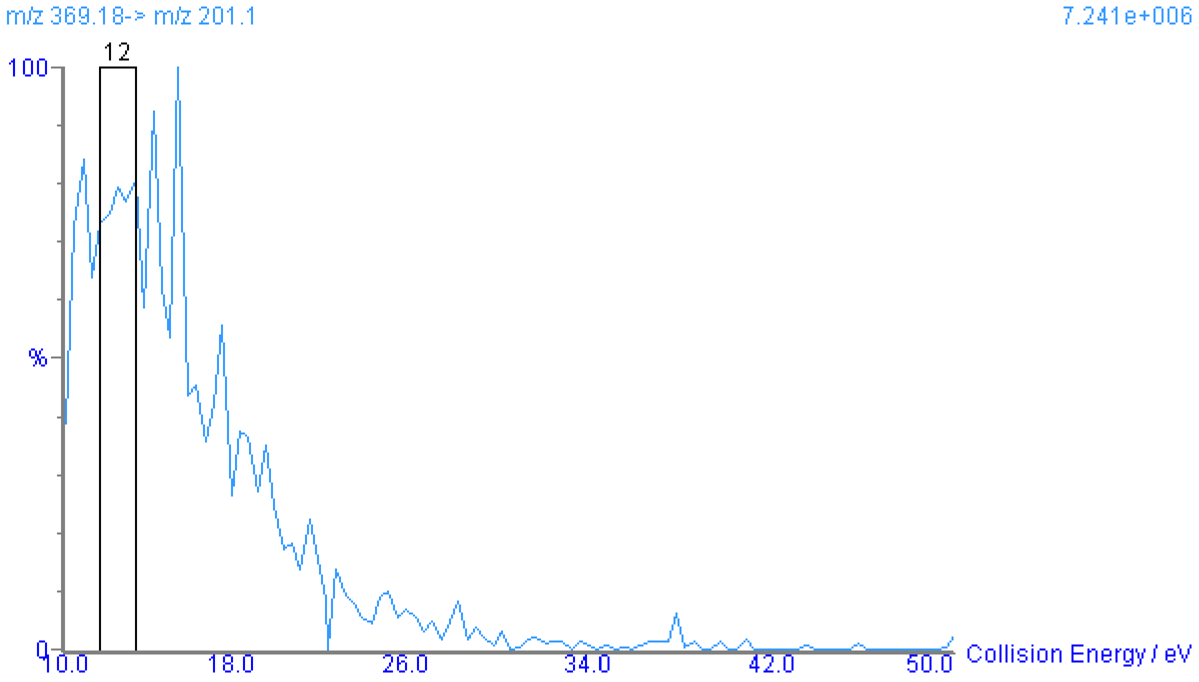

Collision Energy Optimize Spectrum for m/z 369.18 -> 201.1

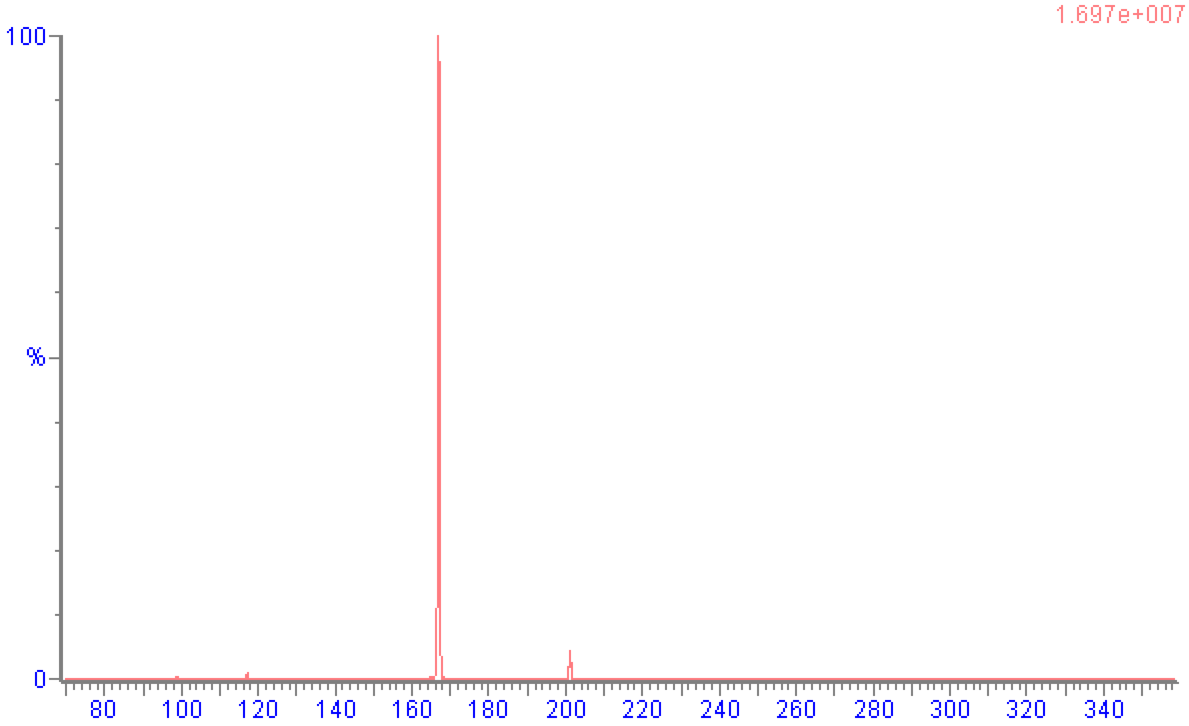

Supplement: S1 Supporting Information — (PDF) [file pone.0198469.s001.pdf]
