# Supplementary material for: Stabilization benefits of single and multi-layer self-nanoemulsifying pellets: A poorly-water soluble model drug with hydrolytic susceptibility
Source: PLoS One. 2018 Jul 19;13(7):e0198469. doi: 10.1371/journal.pone.0198469 (PMC6053139; doi:10.1371/journal.pone.0198469)

AutoTune Report

This report displays the results of the MRM development carried out as part of an autotune.

Calibration method : C:\MassLynx\DEFAULT.PRO\AcquDB\calibration.cal

MSMS Tune method : C:\MassLynx\Cinnarizinne.pro\ACQUDB\CNN deg 385-403.ipr

Daughter search data file : C:\MassLynx\Cinnarizinne.PRO\data\Product.raw

Date: Generated on Mon 26 Mar 2018 at 10:09

Cone Optimize Chromatogram for m/z 385.14

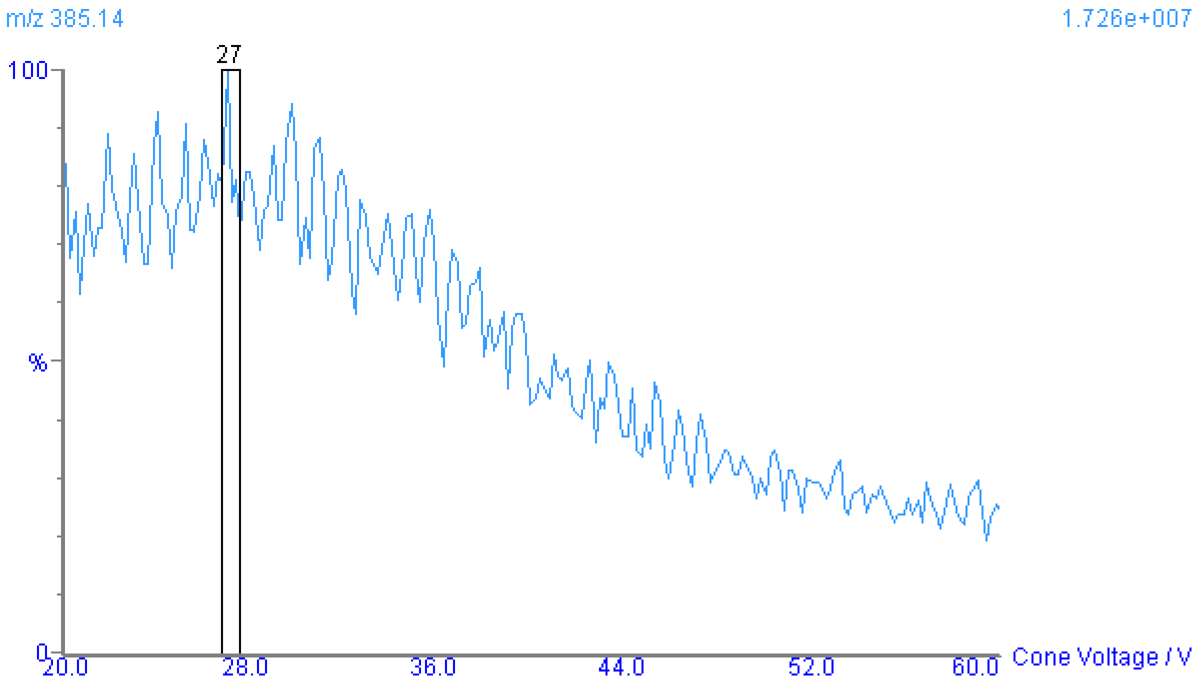

Cone Optimize Spectrum for m/z 385.14

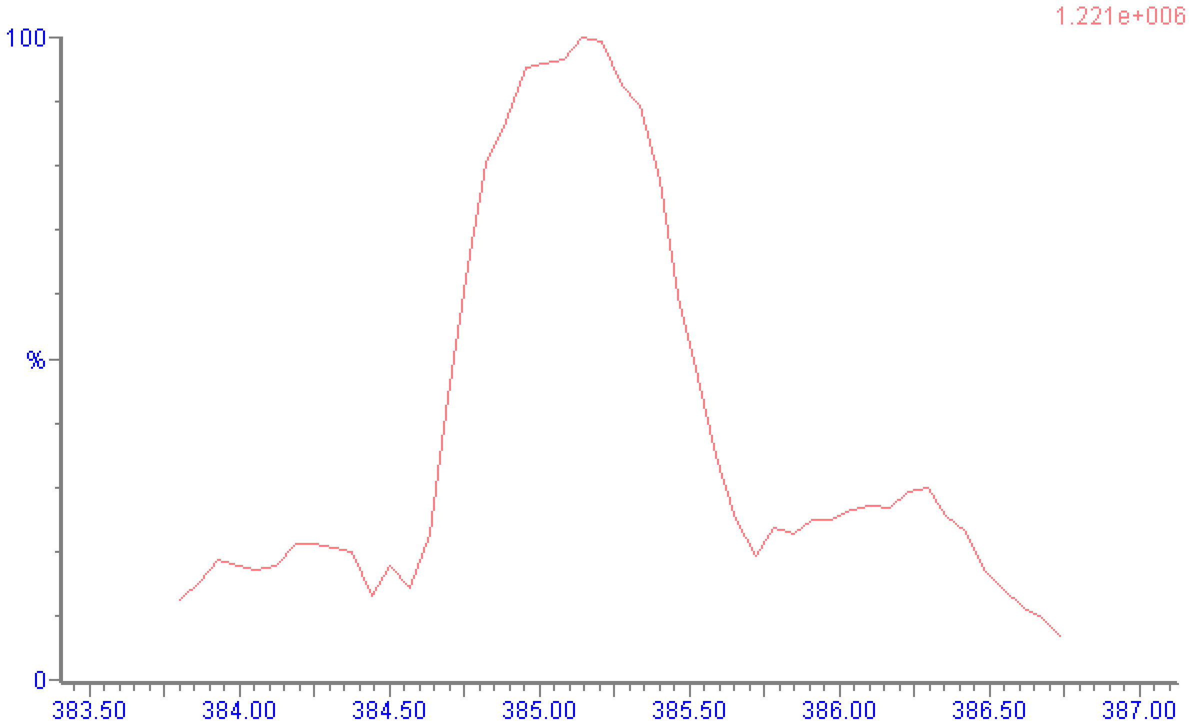

Cone Optimize Spectrum for m/z 385.14

The MRM experiment developed: C:\MassLynx\Cinnarizinne.pro\ACQUDB\CNN Method DEG 385-403.exp

Precursor Mass	Product Mass	Cone Voltage	Collision Energy
385.14	91.0	27.00	50.00
385.14	117.0	27.00	26.00
385.14	167.0	27.00	14.00

Collision Energy Optimize Chromatogram for m/z 385.14 -> 91.0

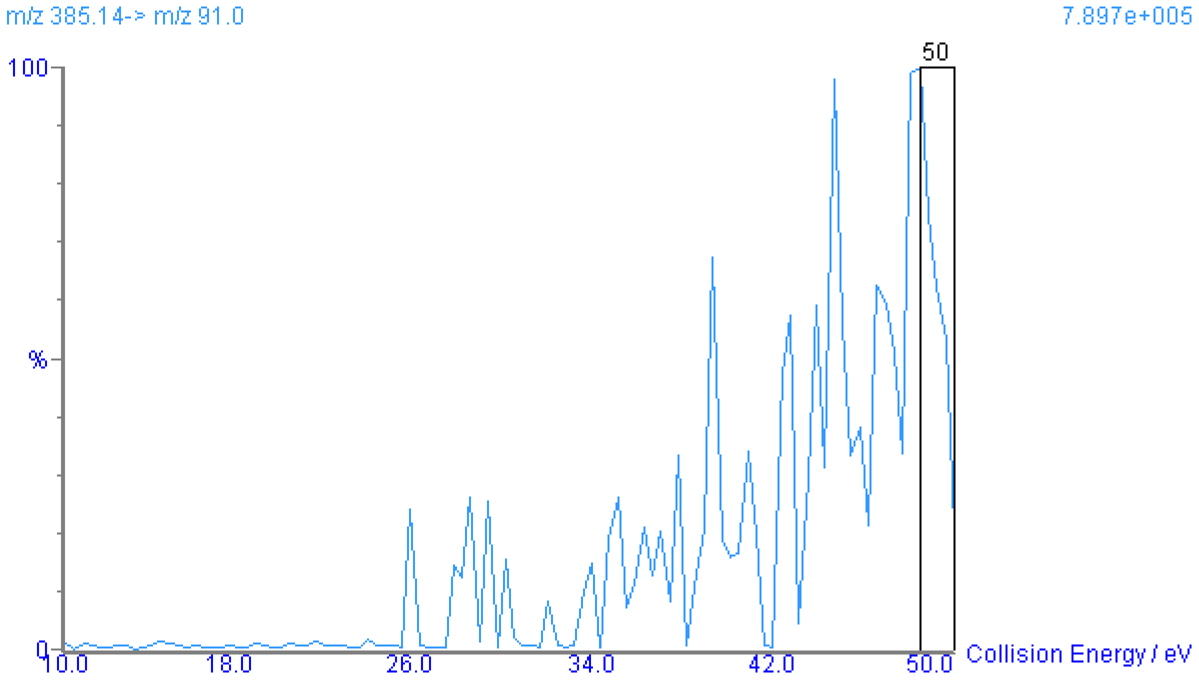

Collision Energy Optimize Spectrum for m/z 385.14 -> 91.0

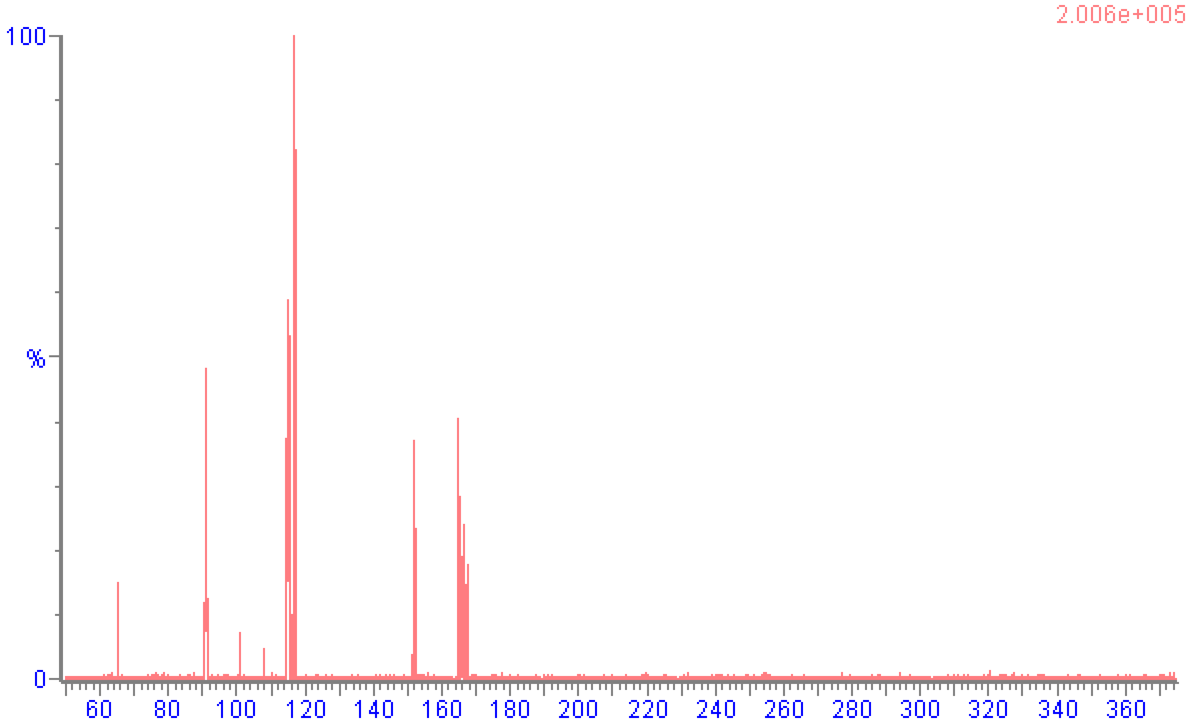

Collision Energy Optimize Chromatogram for m/z 385.14 -> 117.0

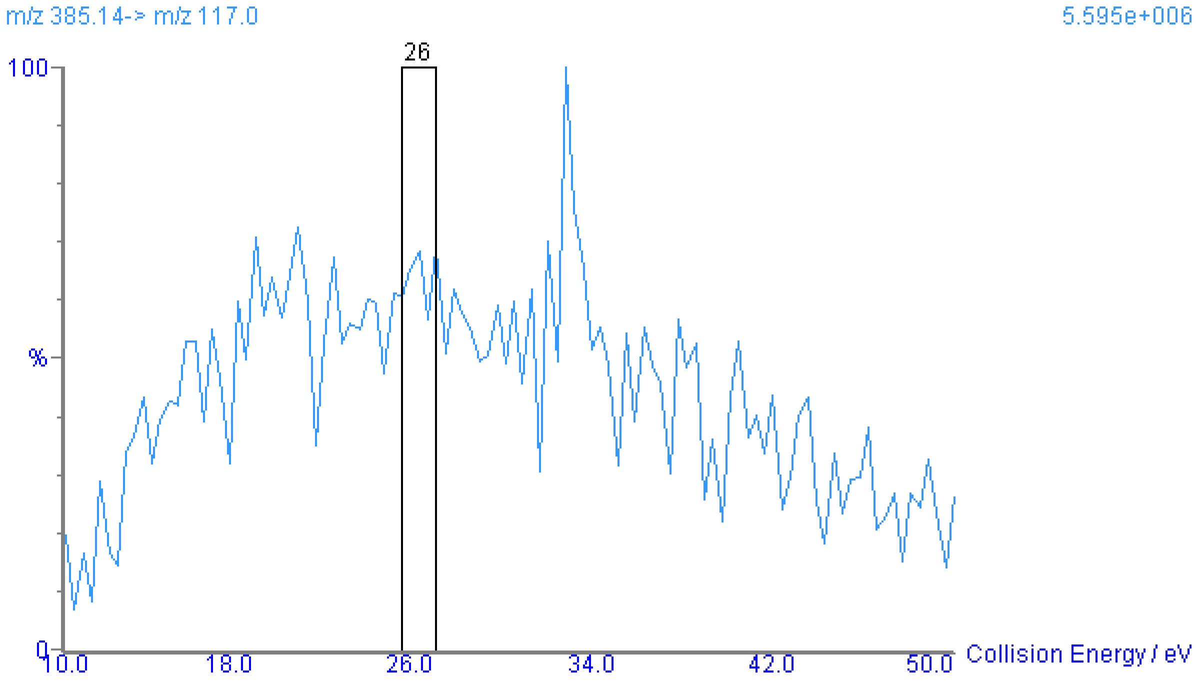

Collision Energy Optimize Spectrum for m/z 385.14 -> 117.0

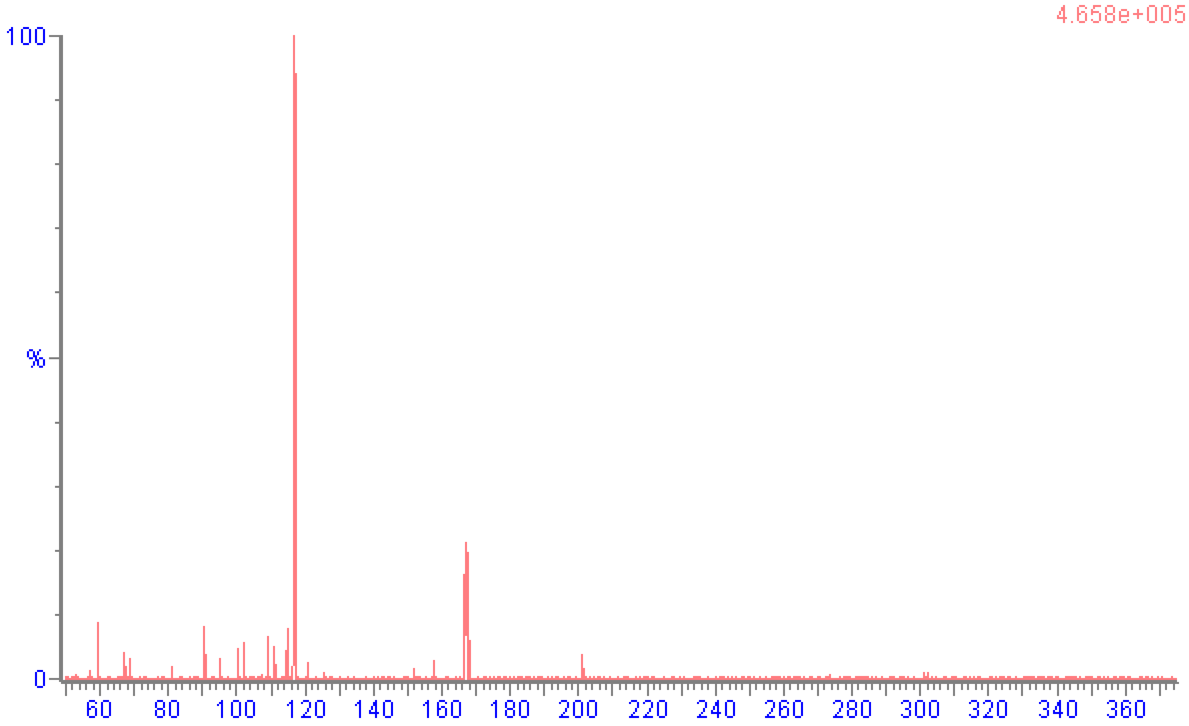

Collision Energy Optimize Chromatogram for m/z 385.14 -> 167.0

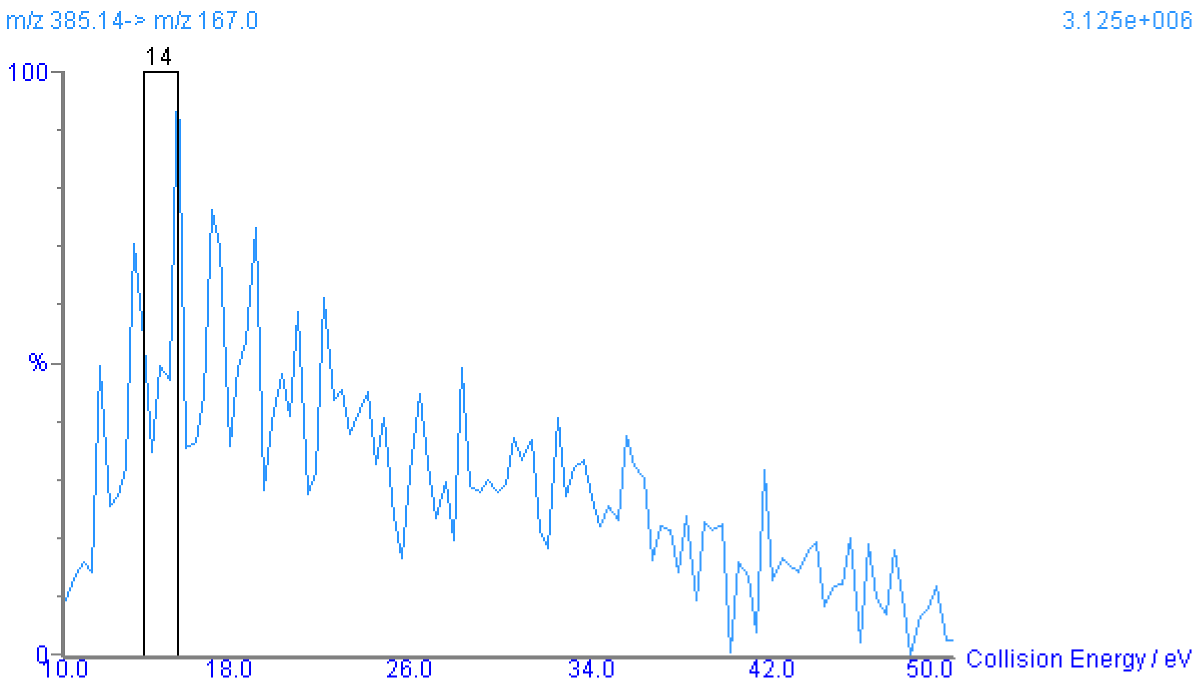

Collision Energy Optimize Spectrum for m/z 385.14 -> 167.0

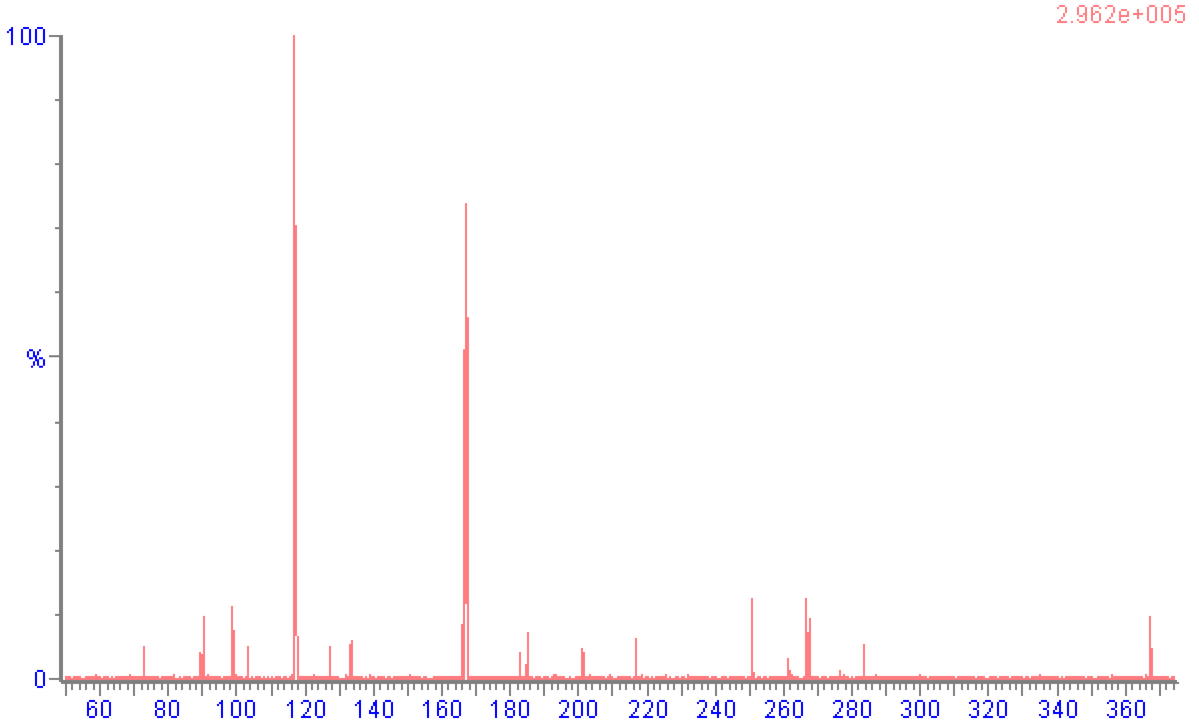

Supplement: S2 Supporting Information — (PDF) [file pone.0198469.s002.pdf]
